# Associations between fundamental movement skills and accelerometer-measured physical activity in Chinese children: the mediating role of cardiorespiratory fitness

**DOI:** 10.7717/peerj.17564

**Published:** 2024-06-24

**Authors:** Dingyi Zhang, Sitong Chen, Fei Xin, Clemens Drenowatz, Xiaoqing Hu, Yan Tang

**Affiliations:** 1 School of Physical Education, Shanghai University of Sport, Shanghai, China; 2 Institute for Health and Sport, Victoria University, Melbourne, Australia; 3 Division of Sport, Physical Activity and Health, University of Education Upper Austria, Linz, Austria; 4 College of Sports Science, Fujian Normal University, Fuzhou, China

**Keywords:** Motor competence, Physical fitness, China, Sex difference

## Abstract

**Background and purpose:**

The associations of fundamental motor skills (FMS), health-related physical fitness (*e.g*., cardiorespiratory fitness, CRF), and moderate-vigorous physical activity (MVPA) have been demonstrated in Western children, but these associations have not yet been validated in a sample of Chinese children. The aims of this study, therefore, were to examine the association between FMS subdomains and MVPA in a sample of Chinese children and to evaluate whether this association is mediated by CRF.

**Methods:**

A cross-sectional study consisting of 311 children aged 8–12 years (49.2% girls; mean age = 9.9 years) from Shanghai was conducted. FMS, CRF and MVPA were assessed using the Test of Gross Motor Development-3rd Edition, Progressive Aerobic Cardiovascular Endurance Run and ActiGraph GT3X accelerometers. Preacher & Hayes’s bootstrap method was used to test the mediating effects of CRF on the association between FMS and MVPA.

**Results:**

CRF fully mediated the association between total FMS and MVPA in girls (indirect effects, *b* = 0.21, 95% CI [0.07–0.37]), while the mediation was only partial in boys (indirect effects, *b* = 0.12, 95% CI [0.01–0.26]). CRF fully mediated the association between locomotor skills and MVPA in girls (indirect effects, *b* = 0.27, 95% CI [0.09– 0.51]), whereas CRF partially mediated the association between object control skills and MVPA in boys (indirect effects, *b* = 0.15, 95% CI [0.18–0.35]).

**Conclusion:**

In order to better design and implement sex-specific interventions aiming to increase MVPA, it is essential to consider FMS subdomains and CRF alongside the sex differences in the association between them.

## Introduction

Sufficient evidence demonstrates that participation in moderate-to-vigorous physical activity (MVPA) is beneficial for children on a variety of health indicators, including enhanced physical fitness levels (Janssen & Leblanc, 2010), improved cognitive benefits (Bao et al., 2024) and reduced mental health problems (Poitras et al., 2016). To reap the additional health benefits, the World Health Organization Physical Activity Guidelines recommend that children should engage in on average 60 min or more of daily MVPA (World Health Organization, 2020). However, the prevalence of meeting the guidelines remains low (Guthold et al., 2020). In China, for example, a survey shows that only 50.0% of children meet the recommended MVPA levels (Liu et al., 2022). In addition, a secular trend study reports a downward trend in physical activity (PA) from 2004 to 2015 in children in China (Yang et al., 2021). In order to increase PA levels in Chinese children, it is imperative to understand the key factors of MVPA.

Fundamental movement skills (FMS) are considered as a significant correlate of MVPA (Britton, Belton & Issartel, 2019; Cohen et al., 2014). FMS are defined as basic learnt movement patterns that do not develop naturally and are suggested to be a critical component for participation in more complex physical and sporting activities (Barnett et al., 2016). They are typically classified into three categories: locomotor skills (*e.g*., jumping, running), object control skills (*e.g*., kicking, catching), and stability skills (*e.g*., balancing, bending) (Barnett et al., 2016). Most of the current studies have focused on the association between locomotor skills and MVPA, as well as the association between object control skills and MVPA (Gu et al., 2021; Holfelder & Schott, 2014; Kaioglou et al., 2022; Xin et al., 2020), rather than the association between stability skills and MVPA, which could be attributed to some studies that have found an either weak or insignificant association between stability skills and MVPA (Iivonen et al., 2013; Nilsen et al., 2019). Given the results indicating that boys are more competent in object control skills whereas girls are more competent in locomotor skills (Barnett et al., 2009; Morgan et al., 2008), it is important to examine the association between FMS subdomains and MVPA separately for boys and girls. Although the study by Hume et al. (2008b) found that object control skills were more strongly related to MVPA in boys while the association with locomotor skills was stronger in girls, another study did not find a sex difference in the association between FMS subdomains and MVPA (Jaakkola et al., 2019). Furthermore, the majority of research on the connection between FMS subdomains and MVPA was carried out in Western nations (Gu et al., 2021; Hume et al., 2008b; Jaakkola et al., 2019). In contrast, a very small number of studies on Chinese children (Chen et al., 2018) were conducted, generating mixed findings. For example, one study indicated that Chinese children’s object control skills predicted pedometer-measured PA, although locomotor skills did not (Han et al., 2022). However, another study suggested that neither object control nor locomotor skills were associated with accelerometer-measured MVPA in Chinese preschoolers (He et al., 2024). Thus, additional research is required to clarify the association between FMS subdomains and MVPA in Chinese boys and girls separately.

To better understand the FMS–PA association and its potential mechanisms, Stodden and colleagues proposed a conceptual model postulating that health-related fitness is an important mediator in the association between FMS and PA (Stodden et al., 2008). This model was first thoroughly tested by a cross-sectional study of Khodaverdi et al. (2016) who investigated the role of health-related fitness as a mediator in the association between FMS and PA in Iranian girls. They found that only cardiorespiratory fitness (CRF) mediated the association between locomotor skills and self-reported PA (Khodaverdi et al., 2016). Based on this finding, CRF is thought to have a stronger association with PA compared to other health-related fitness components (Chen et al., 2018; khodaverdi, Goodway & Stodden, 2017). In the meanwhile, two studies also found the mediating effect of CRF in the association between FMS and PA. A cross-sectional study by Kaioglou et al. (2022) found that CRF fully mediated the association between FMS and pedometer-measured PA, and a longitudinal study by Lima et al. (2017) reported that CRF fully mediated the association between FMS and accelerometer-measured MVPA. Moreover, while some studies have revealed sex differences in the mediating role of health-related fitness in the association between FMS and PA (Jaakkola et al., 2019; Lopes & Rodrigues, 2021), no research has examined sex differences in the mediating role of CRF in the association between specific FMS subdomains and MVPA. Given that previous research has shown sex differences in the associations between FMS subdomains and MVPA (Bryant et al., 2014; Holfelder & Schott, 2014), it is necessary to examine the mediating role of CRF in the association between FMS subdomains and MVPA, separately for boys and girls, enhancing the understanding of the complex association and provide critical information for interventions or planning sports lessons.

The aim of this study, therefore, were to (1) examine the associations among FMS subdomains and accelerometer-measured MVPA in Chinese children, and (2) explore whether CRF mediates the associations between FMS subdomains and MVPA in boys and girls, respectively.

## Materials and Methods

### Participants

A cross-sectional study was conducted in Shanghai, China from April to May 2021. Multistage stratified random sampling was used to recruit participants from four primary schools located in four districts (*i.e*., Yangpu, Jingan, Hongkou, and Jiading). The principals of each participating school consented to take part in this study. One class was randomly selected in grades 3 to 5 from each school, resulting in a total of 12 classes that were invited to participate in this study.

This study was approved by the Institutional Review Board of the Shanghai University of Sport (102772022RT067). Permissions were received from the principals of all participating schools. Written informed consent was signed by parents or guardians, and verbal assent was obtained from all participating children prior to data collection. The consent rate of potential participants was 89.6%. All procedures were conducted in line with the Declaration of Helsinki.

A total of 374 children aged 8–12 years agreed to participate in the present study and provided written informed consent. Among the 374 participants, 63 children were removed from data analysis due to incomplete assessments of FMS (*n* = 20), CRF (*n* = 5), and MVPA (*n* = 38). Accordingly, 311 (49.2% girls; mean age = 9.9 years) participants were included in the final analysis.

### Measures

#### Fundamental movement skills

FMS was assessed by the Test of Gross Motor Development-3rd Edition (TGMD-3) (Ulrich, 2019). The TGMD-3 comprises two subdomains: (1) locomotor skills: run, hop, gallop, skip, slide, and horizontal jump; and (2) object control skills: catch, kick, overhand throw, underhand throw, one handed strike, two handed strike, and stationary dribble(Ulrich, 2019). Each skill was evaluated twice based on three to five performance criteria. For every criterion, children’s performance was scored as 1 (behavioral component presented) or 0 (not presented). The TGMD-3 is a valid and reliable instrument with acceptable psychometric performance, which can be used in Chinese children aged 3–12 years (Li, Wang & Ulrich, 2022).

Before commencing the formal assessments, trained researchers thoroughly explained and demonstrated each skill to the participants. The performances were recorded on video using a Sony HDR-XR500 video camera. Subsequently, video analyses were conducted by two expert evaluators who had previously achieved an inter-rater reliability of at least 90% (ICC ≥ 0.90). The inter-rater reliability scores for the 13 assessed skills ranged from 0.91 to 0.97. Aggregate scores were then calculated for locomotor skills (ranging from 0 to 46), object control skills (ranging from 0 to 54), and the total FMS score (ranging from 0 to 100).

#### Cardiorespiratory fitness

CRF was assessed using the Progressive Aerobic Cardiovascular Endurance Run (PACER) (Meredith & Welk, 2010), which is a reliable and valid test for children (Morrow, Martin & Jackson, 2010). Participants were required to continuously run back and forth between two parallel lines with a 20 m distance in-between within a pre-set time limit. The participants’ pace was dictated by an audio signal that gradually accelerated. Initially, the running pace was set at 8.5 km/h, with an increment of 0.5 km/h every minute. Before the test commenced, each participant engaged in a warm-up session and observed a demonstration conducted by the primary researcher. Instructions were clear: participants were to run in straight lines and ensure that one foot touched the 20-m mark just prior to each signal beep. The test concluded when a participant either voluntarily ceased due to exhaustion or failed to meet the 20-m mark for two consecutive laps. The number of laps completed by each child was meticulously recorded by the lead author and three experienced researchers throughout the duration of the test.

#### Moderate-to-vigorous physical activity

MVPA was measured using triaxial accelerometers (ActiGraph GT3X; ActiGraph LLC, Pensacola, FL, USA) whose reliability and validity have been shown in children (Robusto & Trost, 2012). Two researchers were responsible for distributing and collecting numbered accelerometers in each classroom. Each participating child was equipped with an accelerometer, which they were instructed to wear during waking hours for seven consecutive days. The children were directed to remove the devices only for bathing, sleeping, or swimming. Consistent with methodologies in similar studies (Lima et al., 2017), the minimum required duration for accelerometer data inclusion was established as 4 days, comprising 3 weekdays and 1 weekend day. Non-wear time was defined as at least 60 min of consecutive zero counts (Ainsworth et al., 2011). A valid day was defined by at least 10 h of wear time during weekdays and weekend days. After the test, the original accelerometer data was downloaded to a personal computer using the ActiLife 6.5.2 software (ActiGraph LLC, Pensacola, FL, USA). The sampling interval (epoch) in the present study was set at 1 s (Trost, McIver & Pate, 2005). The cut-off points for Chinese children established by Zhu, Chen & Zhuang (2013) were used to define intensities of MVPA: ≥ 2,800 counts per minute for MVPA.

#### Covariates

Weight and height were measured to the nearest 0.1 kg and 0.1 cm, respectively with participants wearing light clothing and being barefoot. Both measures were assessed using a height-weight scale (GFMSS-IV; Jianmin, Beijing, China). Body mass index (BMI) was calculated with weight (kg)/height (m)².

#### Procedure

Data collection was conducted by four trained graduate students specializing in Kinesiology, adhering to a predefined protocol to minimize the risk of data contamination. In the initial testing week at each school, MVPA data were measured over a 7-day period, encompassing five weekdays and two weekend days. Subsequently, FMS assessments were conducted during the first two Physical Education class sessions of the second week. In the remaining Physical Education session that week, BMI and CRF measurements were taken.

#### Statistical analysis

All data analyses were performed using SPSS version 24.0 (IBM Corp., Armonk, NY, USA) for Windows. Before the analyses, data were checked for outliers, missing values, and normality. Descriptive statistics (M ± SD) were calculated for FMS, MVPA and CRF. Pearson correlation coefficients were calculated to determine the association between FMS, CRF, and MVPA for boys and girls, separately. The strength of the correlations was interpreted using Cohen’s (2013) criteria as low (*r/β* = 0.10–0.29), moderate (*r/β* = 0.30–0.49), or high (*r/β* ≥ 0.50).

To examine the mediating role of CRF in the association between children’s FMS and PA, the PROCESS-Macro function was used (version 3.5). Mediator analyses were performed for boys and girls, separately, because the mediation effects of CRF have been shown to vary by sex (Jaakkola et al., 2019; Lopes & Rodrigues, 2021). Given the associations of age and BMI with FMS, PA, and CRF (He et al., 2024; Xin et al., 2020), those two variables were included as covariates in the mediation analysis.

For the mediation analysis, CRF was identified as a mediator when it met the following conditions: (1) the independent variable (FMS) was significantly correlated with the dependent variable (MVPA), (2) the independent variable (FMS) was significantly correlated with the mediator (CRF), (3) CRF was significantly correlated with the dependent variable (MVPA), (4) the association between the independent variable (FMS) and dependent variable (MVPA) became insignificant (full mediation) or its coefficient weakened (partial mediation) after adding CRF (Baron & Kenny, 1986). Bootstrapping was used to test the significance of the mediating effect, with confidence intervals (95% CI) generated from 5,000 bootstrap samples (Hayes, 2013). Mediation effects were considered significant if zero was not between the upper and lower bounds of the bootstrap 95% confidence interval (Hayes, 2013). The statistical significance was set at *p* < 0.05.

## Results

Sample characteristics and means of FMS, CRF, and MVPA are presented by sex in Table 1. Boys outperformed girls in FMS, object control skills and MVPA (all *p* < 0.01). Conversely, there were no sex differences in locomotor skills and C RF (both *p* > 0.05).

**Table 1:** Sample characteristics of study participants by sex (*N* = 311).

	Girls (*n* = 153)		Boys (*n* = 158)		Sex difference
	**Mean**	**SD**	**Mean**	**SD**	***p***
Age	9.95	1.06	10.03	0.94	0.531
BMI	17.41	3.63	19.13	3.25	**<0.001**
FMS (raw score)	71.30	6.97	73.70	8.01	**<0.01**
LS (raw score)	36.27	4.29	35.55	4.83	0.168
OCS (raw score)	35.03	4.73	38.15	4.81	**<0.001**
CRF (lap)	20.18	7.47	21.64	8.74	0.116
MVPA (min/day)	31.03	11.58	37.93	13.47	**<0.001**

**Notes:**

SD, standard deviation; BMI, Body Mass Index; FMS, fundamental movement skills; LS, locomotor skills; OCS, object control skills; CRF, cardiorespiratory fitness; MVPA, moderate-to-vigorous physical activity; *p* values with bold type indicated *p* < 0.05.

Correlations among FMS, CRF and MVPA in the study participants by sex are shown in Table 2. There was a moderate correlation between FMS and CRF in both sexes (for girls, *r* = 0.49, *p* < 0.01; for boys, *r* = 0.41, *p* < 0.01). Locomotor skills were significantly associated with MVPA in girls (*r* = 0.19, *p* < 0.05), while object control skills were significantly associated with MVPA in boys (*r* = 0.20, *p* < 0.05). The correlation between CRF and MVPA was also low in both sexes (for girls, *r* = 0.23, *p* < 0.01; for boys, *r* = 0.24, *p* < 0.01).

**Table 2:** Pearson’s correlation coefficient between key study variables by sex.

	FMS	LS	OCS	CRF
Girls (*n* = 153)				
LS	0.75**			
OCS	0.80**	0.19*		
CRF	0.49**	0.41**	0.35**	
MVPA	0.14	0.19*	0.03	0.23**
Boys (*n* = 158)				
LS	0.83**			
OCS	0.83**	0.38**		
CRF	0.41**	0.38**	0.31**	
MVPA	0.19*	0.11	0.20*	0.24**

**Notes:**

FMS, fundamental movement skills; LS, locomotor skills; OCS, object control skills; CRF, cardiorespiratory fitness; MVPA, moderate-to-vigorous physical activity; *p < 0.05; **p < 0.01.

**Figure 1:**
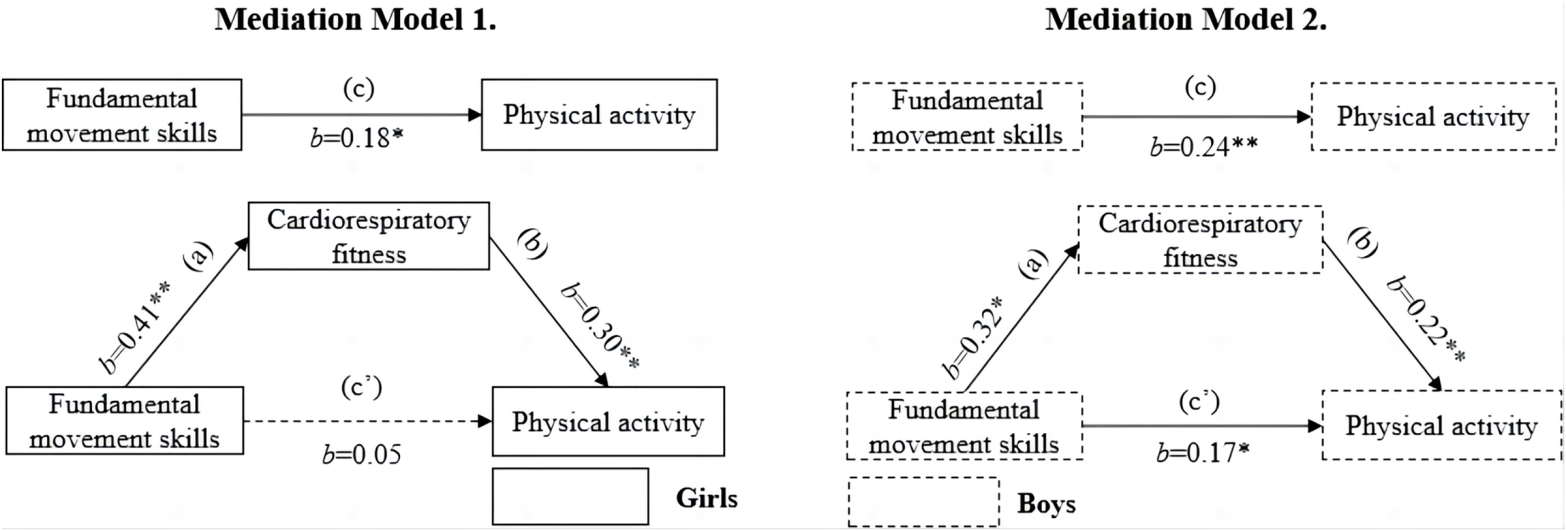
Mediating roles of cardiorespiratory fitness in the association between fundamental movement skills and moderate‐to‐vigorous physical activity.

Table 3 shows the total, direct and mediated effect values for all mediation models. Figure 1 displays the results of the mediation models for the association between FMS and MVPA. It was found that FMS was positively associated with MVPA in both sexes (model 1 for girls, *b* = 0.18, *p* < 0.05; model 2 for boys, *b* = 0.24, *p* < 0.01). In girls, the association between FMS and MVPA was fully mediated by CRF, as there was no significant direct association between FMS and MVPA (*b* = 0.05, *p* > 0.05) after adding CRF. CRF partially mediated the association between FMS and MVPA in boys, representing approximately 29.3% of the total effect. Despite the significant associations between FMS and CRF (*b* = 0.32, *p* < 0.05) as well as CRF and MVPA (*b* = 0.22, *p* < 0.01), the association between FMS and MVPA remained significant after adding CRF (*b* = 0.17, *p* < 0.05).

**Table 3:** Bootstrap analysis of mediating effects of models.

		Effect	SE	Lower	Upper	*p*
Model 1	Total effect	0.29	0.14	0.04	0.02	<0.05
	Direct effect	0.08	0.14	−0.21	0.38	>0.05
	Indirect effect	0.21	0.07	0.07	0.37	<0.05
Model 2	Total effect	0.41	0.14	0.13	0.67	<0.05
	Direct effect	0.29	0.14	0.01	0.57	<0.05
	Indirect effect	0.12	0.06	0.01	0.26	<0.05
Model 3	Total effect	0.56	0.22	0.12	0.99	<0.05
	Direct effect	0.29	0.23	−0.16	0.75	>0.05
	Indirect effect	0.27	0.11	0.09	0.51	<0.05
Model 4	Total effect	0.38	0.23	−0.06	0.83	>0.05
	Direct effect	0.19	0.23	−0.28	0.64	>0.05
	Indirect effect	0.19	0.09	0.04	0.42	<0.05
Model 5	Total effect	0.16	0.21	−0.25	0.56	>0.05
	Direct effect	−0.07	0.21	−0.47	0.34	>0.05
	Indirect effect	0.23	0.09	0.07	0.42	<0.05
Model 6	Total effect	0.69	0.22	0.26	0.94	<0.05
	Direct effect	0.54	0.22	0.09	0.98	<0.05
	Indirect effect	0.15	0.08	0.18	0.35	<0.05

**Figure 2:**
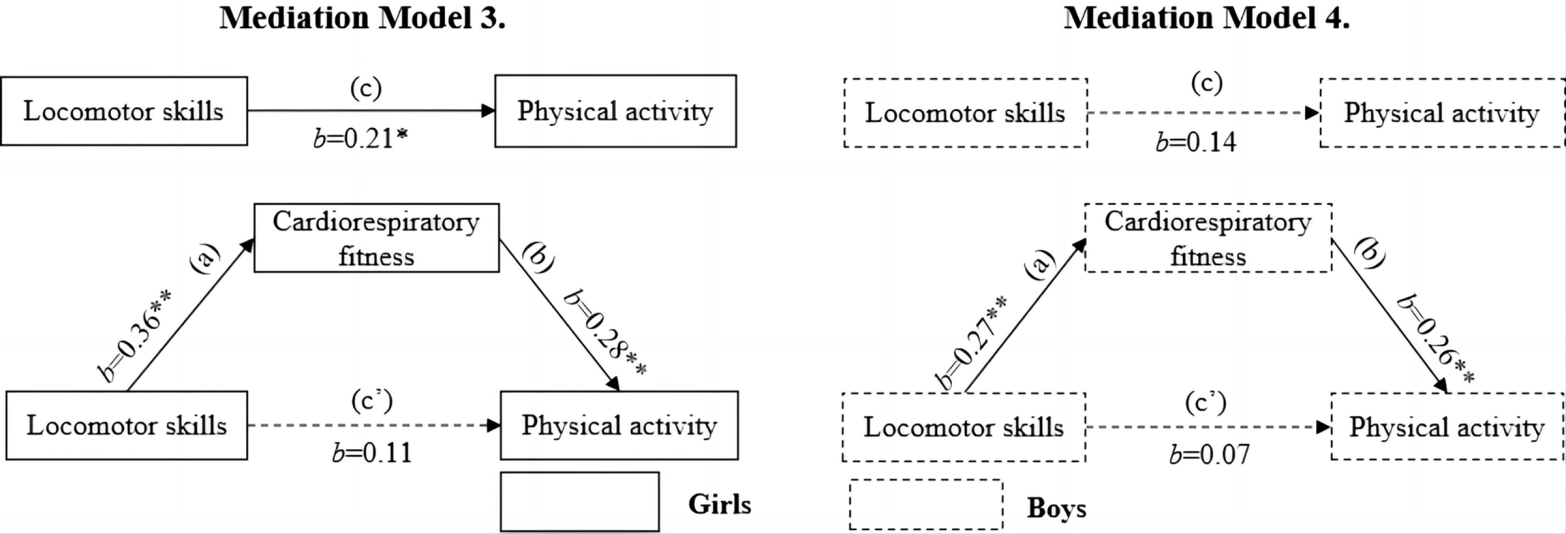
Mediating roles of cardiorespiratory fitness in the association between locomotor skills and moderate‐to‐vigorous physical activity.

Figure 2 displays the results of the mediation models for the association between locomotor skills and MVPA. It was found that locomotor skills was positively associated with MVPA in girls but not in boys (model 3 for girls, *b* = 0.21, *p* < 0.05; model 4 for boys, *b* = 0.14, *p* > 0.05). In boys, CRF failed to mediate the association between locomotor skills and MVPA. In girls, the association between locomotor skills and MVPA was fully mediated by CRF, as there was no significant direct association between locomotor skills and MVPA (*b* = 0.11, *p* > 0.05) after adding CRF.

**Figure 3:**
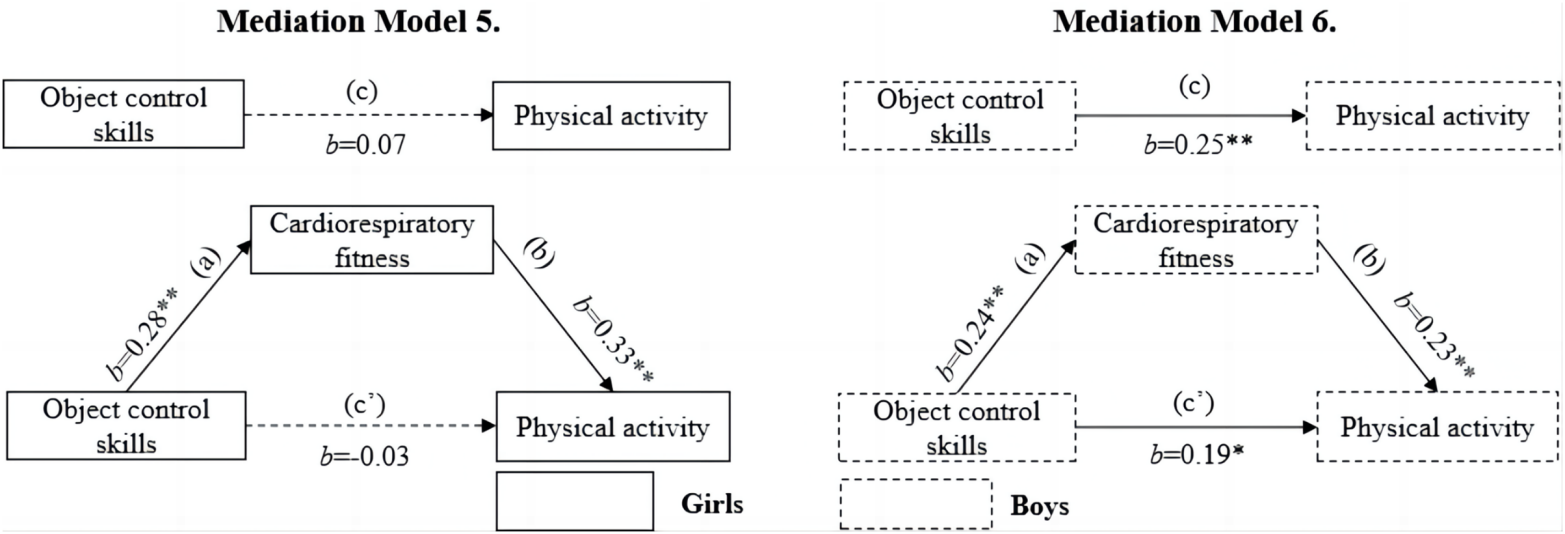
Mediating roles of cardiorespiratory fitness in the association between object control skills and moderate‐to‐vigorous physical activity.

Figure 3 displays the results of the mediation models for the association between object control skills and MVPA. It was found that object control skills was positively associated with MVPA in boys but not in girls (model 5 for girls, *b* = 0.07, *p* > 0.05; model 6 for boys, *b* = 0.25, *p* < 0.01). In girls, CRF failed to mediate the association between object control skills and MVPA. CRF partially mediated the association between object control skills and MVPA in boys, representing approximately 21.7% of the total effect. There was a significant association between object control skills and CRF (*b* = 0.24, *p* < 0.01) as well as CRF and MVPA (*b* = 0.23, *p* < 0.01) in boys, there remained a significant direct association between object control skills and MVPA (*b* = 0.19, *p* < 0.05) after adding CRF.

## Discussion

The aim of this study was to investigate the associations among FMS, CRF and MVPA in Chinese children. The results indicate that there are sex differences in the association between FMS subdomains and MVPA, as well as in the mediating role of CRF between them. This is one of the very few studies exploring the association between FMS, CRF and MVPA in Chinese children, and one of very few indicating the sex differences in these associations. In conclusion, the findings emphasize the necessity of enhancing specific FMS subdomains and CRF to promote MVPA participation in both sexes of children.

Regarding the associations between FMS subdomains and MVPA, our findings revealed the sex difference, with object control skills and locomotor skills being associated with MVPA only in boys and girls, separately. Our findings can be supported by two previous studies. Hume et al. (2008a) reported that object control skills were more strongly related to MVPA in boys, while locomotor skills were related to MVPA in girls. Bryant et al. (2014) reported that boys’ catching (*i.e*., object control skills) was a predictor for their total PA, whereas girls’ hopping and jumping (*i.e*., locomotor skills) were predictive of their total PA 1 year later. Such sex differences may be attributed to a greater likelihood of boys’ participation in object control skills-related sports, such as basketball, soccer and baseball, whereas girls engage more in locomotor skills-related sports (*e.g*., dancing, jogging, gymnastics) (Dudley et al., 2018; Van Rheenen, 2012). Given the sex-specific associations between FMS subdomains and MVPA and that childhood is a critical period for the development of FMS, targeted interventions for FMS are needed to promote children’s MVPA. For example, interventions for boys should focus more on object control skills such as throwing, kicking, and striking, while girls-oriented programs are advised to emphasize locomotor skills such as running, hopping, and galloping (Morgan et al., 2008).

In addition to examining the direct association between FMS and MVPA, this study investigated the mediating effect of CRF on the association between overall FMS and MVPA in boys and girls, separately. Stodden et al. (2008) demonstrated that the development of FMS is a causal mechanism to promote either positive or negative trajectories of health-related fitness (*e.g*., CRF) and PA. Although previously longitudinal (Lima et al., 2017) and cross-sectional (Kaioglou et al., 2022) studies confirmed the role of CRF as a mediator in the association between total FMS and PA, these studies have failed to examine these associations separately for boys and girls. We found that girls’ CRF fully mediated the association between total FMS and MVPA, while boys’ CRF partially mediated the association between total FMS and MVPA. Although some studies have examined the mediating role of health-related fitness rather than CRF in the association between FMS and PA, they could still be used to in part explain our findings. A cross-sectional study conducted by Jaakkola et al. (2019) reported that health-related fitness was a mediator between total FMS and accelerometer-measured MVPA, with a partial mediation in Finnish boys and a full mediation in girls. Also, a cross-sectional study by Lopes & Rodrigues (2021) found that health-related fitness had a fully mediating effect on total FMS and self-reported PA in girls and a partially mediating effect on the association between total FMS and self-reported PA in boys. The above findings imply that the mediating effect of health-related fitness (*e.g*., CRF) on the association between FMS and PA may be stronger in girls compared to boys. As the model of Stodden et al. (2008) did not consider the sex differences in the associations among FMS, health-related fitness, and PA in children and adolescents, our results are meaningful to empirically improve the model.

Interestingly, our results indicate that CRF fully mediated the locomotor skills-MVPA association in girls, whereas CRF partially mediated the object control skills-MVPA association in boys. According to the findings of Khodaverdi et al.’s (2016) cross-sectional study, CRF mediated the association between locomotor skills and self-report PA in girls, while it failed to mediate the association between object control skills and PA in girls. However, their study did not include boys. This discrepancy of skill-specific and sex differences may be due to different behavioral patterns in boys and girls and their associations with object control skills and locomotor skills (Hume et al., 2008a), as well as locomotor skills being more strongly associated with CRF in children than object control skills (Khodaverdi et al., 2016; Williams et al., 2008). Specifically, although activities involving locomotor skills (*e.g*., dancing, jogging) and object control skills (*e.g*., basketball, soccer) are generally associated with the repetitive movement that enhances CRF (Cattuzzo et al., 2016), proficiency in object control skills is associated with multiple components of health-related fitness (*e.g*., muscle strength is important for jumping in basketball), not just CRF (Britton, Belton & Issartel, 2019). In other words, apart from CRF, it is possible that other components of health-related fitness, such as muscle strength, may also in part play a role in mediating object control skills and MVPA in boys. Given the scarce information available in the literature, future research is needed to examine the mediating effects of health-related fitness components (*e.g*., muscle strength and CRF) in the association between FMS subdomains (*e.g*., locomotor skills and object control skills) and MVPA. This information is useful because it will allow us to understand the mechanisms of MVPA participation and the model of Stodden et al., (2008) that differ between sexes and, as a result, enable us to design more appropriate interventions to promote MVPA levels in both sexes.

### Strength and limitation

This study has some limitations. First, due to the cross-sectional design of our study, causal inferences regarding the associations between key variables are speculative. Future studies need to use a longitudinal design to examine the mediating role of CRF in the association between FMS subdomains and MVPA to determine the causal nature of these associations. Second, only CRF has been tested rather than all components of health-related fitness in this study, even though many researchers have chosen to investigate the association between FMS, PA, and CRF (Gu, Thomas & Chen, 2017; Kaioglou et al., 2022; Lima et al., 2017). Third, the participants comprised students from four primary schools in Shanghai, thus failing to represent the children from other schools and areas of China completely. Future studies could expand the research scope by employing a large and diverse sample. In addition, although locomotor skills and object control skills were included in this study, it failed to include stability skills. Finally, age and BMI were included as covariates in this study. While BMI is a commonly used alternative body composition measure, it is not always accurate in differentiating between children’s adipose tissue and lean body mass.

Despite these limitations of this study, there are several strengths. The main strength of our study is that it is one of the few studies to investigate the mediating effect of CRF on the association between FMS subdomains and MVPA, along with potential sex differences in these associations. Second, this is one of the few Chinese studies that have explored the association between FMS, CRF and MVPA, as most of the studies of the association between these variables have been based on children in Western countries. Moreover, while previous studies have more often used questionnaires (Khodaverdi et al., 2016; Lopes & Rodrigues, 2021) or pedometers (Kaioglou et al., 2022) to measure PA, we used the accelerometer to assess MVPA.

## Conclusions

Inadequate PA can have a negative impact on children’s health and well-being. Although many studies have shown that FMS and CRF are important variables associated with PA participation, few studies have investigated into the association of these variables in Chinese children, as well as sex differences in the direct and indirect associations of these variables. The present study demonstrated that FMS and CRF were significant correlates of MVPA in Chinese children, and the direct and indirect association between specific FMS subdomains and MVPA differed by sex. More specifically, we discovered that locomotor skills correlated with MVPA in girls, whereas object control skills was associated with MVPA in boys. CRF fully mediated the association between locomotor skills and MVPA in girls, but only partially mediated the association between object control skills and MVPA in boys. These findings provide supportive information for a better understanding of the pathways presented in the theoretical model proposed by Stodden et al. (2008). Based on these findings, increasing levels of CRF and improving specific FMS subdomains in both sexes is essential to increase MVPA in children.

## Supplemental Information

10.7717/peerj.17564/supp-1 Supplemental Information 1 Codebook.

10.7717/peerj.17564/supp-2 Supplemental Information 2 TGMD-3 Test Privileges.

10.7717/peerj.17564/supp-3 Supplemental Information 3 PACER (20_m_shuttle_run) Test Privileges.

10.7717/peerj.17564/supp-4 Supplemental Information 4 Raw_measurements.

10.7717/peerj.17564/supp-5 Supplemental Information 5 Raw_measurements.

10.7717/peerj.17564/supp-6 Supplemental Information 6 STROBE checklist.

## Abbreviations

PA: Physical Activity
MVPA: Moderate-to-Vigorous Physical Activity
FMS: Fundamental Movement Skills
CRF: Cardiorespiratory Fitness
BMI: Body Mass Index
TGMD-3: Test of Gross Motor Development-3rd Edition
M: Mean
SD: Standard Deviation
CI: Confidence Interval
ICC: Intra-class Correlation Coefficient.

## Additional Information and Declarations

### Data Availability

The following information was supplied regarding data availability: The raw measurements are available in the Supplemental Files.
