# Supplementary material for: Associations between fundamental movement skills and accelerometer-measured physical activity in Chinese children: the mediating role of cardiorespiratory fitness
Source: PeerJ. 2024 Jun 24;12:e17564. doi: 10.7717/peerj.17564 (PMC11210481; doi:10.7717/peerj.17564)
Supplement: Supplemental Information 1 [file peerj-12-17564-s001.docx]

| Sex | 1=Boys, 2=Gilrs |
| --- | --- |
| Age | 1=7 years old, 2=8 years old, 3=9 years old, 4=10 years old, 5=11 years old, 6=12 years old |
| Height | cm |
| Weight | kg |
| BMI | BMI = Weight (kg) ÷ (Height (m))^2 |
| MVPA | moderate‐to‐vigorous physical activity **(**min/day) |
| LMS | locomotor skills (raw) |
| OCS | object control skills(raw) |
| CRF | cardiorespiratory fitness(lap) |
| FMS | fundamental movement skills(raw) |
