# Supplementary material for: Associations between fundamental movement skills and accelerometer-measured physical activity in Chinese children: the mediating role of cardiorespiratory fitness
Source: PeerJ. 2024 Jun 24;12:e17564. doi: 10.7717/peerj.17564 (PMC11210481; doi:10.7717/peerj.17564)
Supplement: Supplemental Information 2 [file peerj-12-17564-s002.pdf]

*Note:* This article will be published in a forthcoming issue of the *Journal of Motor Learning and Development*. The article appears here in its accepted, peer-reviewed form, as it was provided by the submitting author. It has not been copyedited, proofed, or formatted by the publisher.

**Section:** Article

**Article Title:** Evaluation of the Psychometric Properties of the Test of Gross Motor Development – 3rd Edition

**Authors:** Elizabeth K. Webster and Dale A. Ulrich

**Affiliations:** School of Kinesiology at Louisiana State University, Baton Rouge, LA.

**Running Head:** TGMD-3 Psychometric Properties

**Journal:** *Journal of Motor Learning and Development*

**Acceptance Date:** October 26, 2016

©2017 Human Kinetics, Inc.

**DOI**: http://dx.doi.org/10.1123/jmld.2016-0003

“Evaluation of the Psychometric Properties of the Test of Gross Motor Development – 3rd Edition”
by Webster EK, Ulrich DA
*Journal of Motor Learning and Development*
© 2017 Human Kinetics, Inc.

Webster is with the School of Kinesiology at Louisiana State University, Baton Rouge, LA.

Address author correspondence to Elizabeth Webster at kipwebster@lsu.edu.

Webster and Ulrich

TGMD-3 Psychometric Properties

http://dx.doi.org/10.1123/jmld.2016-0003

Elizabeth K. Webster

Louisiana State University

Dale A. Ulrich

University of Michigan

Downloaded by Univ Southern Qld on 02/28/17, Volume 0, Article Number 0

"Evaluation of the Psychometric Properties of the Test of Gross Motor Development – 3rd Edition"
by Webster EK, Ulrich DA
*Journal of Motor Learning and Development*
© 2017 Human Kinetics, Inc.

**Abstract**

With recent revisions, the evaluation of the reliability and validity of the Test of Gross Motor Development – 3rd edition (TGMD-3) is necessary. The TGMD-3 was administered to 807 children (*M* age = 6.33 ± 2.09 years; 52.5% male). Reliability assessments found correlations with age were moderate to large; ball skills having a higher correlation ($r = 0.47$) compared to locomotor skills ($r = 0.39$). Internal consistency was very high in each age group and remained excellent for all racial/ethnic groups and both sexes. Test-retest reliability had high ICC agreements for the locomotor (ICC = 0.97), ball skills (ICC = 0.95), and total TGMD-3 (ICC = 0.97). For validity measures, the TGMD-3 had above acceptable item difficulty (range = 0.43-0.91) and item discrimination values (range = 0.34 - 0.67). EFA supported a one-factor structure of gross motor skill competence for the TGMD-3 with 73.82% variance explained. CFA supported the one-factor model ($\chi^2(65) = 327.61$, $p< .001$, CFI = .95, TLI = .94, RMSEA = .10) showing acceptable construct validity for the TGMD-3. Preliminary results show the TGMD-3 exhibits high levels of validity and reliability, providing confidence for the usage and collection of new norms.

Downloaded by Univ Southern Qld on 02/28/17, Volume 0, Article Number 0

"Evaluation of the Psychometric Properties of the Test of Gross Motor Development – 3rd Edition"
by Webster EK, Ulrich DA
*Journal of Motor Learning and Development*
© 2017 Human Kinetics, Inc.

Downloaded by Univ Southern Qld on 02/28/17, Volume 0, Article Number 0

The acquisition of fundamental motor skills (FMS) is a critical component of early childhood. FMS, also known as gross motor skills, are basic, goal-directed movement forms that may be combined and applied to more context-specific skills (Burton & Miller, 1998; Clark, 1994). These FMS enable children to actively engage with their environment and peers, allowing for diverse experiences that contribute to a well-rounded developmental trajectory (Burton & Miller, 1998; Clark, 1994). Components of motor development are continuously improving and being modified in a developing child; this has cascading effects within the cognitive, physical, neuromuscular, and physiological realms, indicating a critical area of examination (Malina, Bouchard, Bar-Or, 2004; Piek, Dawson, Smith, & Gasson, 2008). By examining these skills one may identify delays, monitor progress, or plan activities to improve a child's motor competency (Burton & Miller, 1998; Ulrich, 2000).

Ample research indicates that accruing a multiplicity of FMS sets the foundation for future movements and allows for translation to lifelong physical activity pursuits (Burton & Miller, 1998; Clark & Metcalfe, 2002; Stodden et al., 2008; Robinson et al., 2015). A dynamic and positive relationship has been posited for FMS competence and physical activity participation and engagement in early childhood through adolescence (Logan, Webster, Getchell, Pfieffer, & Robinson, 2015; Stodden et al., 2008; Robinson et al., 2015). Stodden and colleagues (2008) postulated that motor competence, physical activity, perceived motor competence, and health-related fitness all work together throughout the lifespan to lead children into a positive spiral of engagement (i.e., higher physical activity, motor competence, perceived competence, and fitness) or a negative spiral of engagement (i.e., lower physical activity, motor competence, perceived competence, and fitness). More work has been conducted in recent years and have highlighted a positive relationship between physical activity and perceived competence, as well as motor

© 2017 Human Kinetics, Inc.

competence with physical activity, health-related fitness, and inversely with weight status (Robinson et al., 2015).

In measuring motor competency, FMS may be examined by either process-oriented or product-oriented means. The product-oriented approach focuses on the end result of a movement; for example, throwing a ball a specified distance or time to complete a 40-yard dash (Haywood & Getchell, 2014). A process-oriented approach examines the descriptive characteristics, form, or mechanics of a movement; for example arms moving in opposition to legs with elbows bent during running or an elongated step prior to kicking a stationary ball (Haywood & Getchell, 2014; Ulrich, 2000). There are several assessments that examine FMS in young children that utilize either approach of describing movement. The Test of Gross Motor Development (TGMD; Ulrich, 1985; Ulrich, 2000) is one process-oriented assessment, with a few product-oriented components that is widely used for young children. A few examples of product-oriented components included in the TGMD-3 are completing four consecutive gallops, hops, and skips, or throwing a ball underhand at least 15 feet.

The TGMD recently went through a revision to accommodate potential changes in the normative population, as well as to incorporate recommendations from experts in the field of motor development and practitioners who frequently use the assessment to create the TGMD-3 (Ulrich, 2014). Changes included dropping two skills from the TGMD-2, the leap and underhand roll, and replacing these with the skip, underhand throw, and one-hand strike in the TGMD-3. Minor scoring clarifications and wording were adjusted to reflect to reduce ambiguity on scoring for researchers, teachers, and practitioners. The TGMD is comprised of two subscales, locomotor and ball skills (i.e., object control skills), that evaluate six to seven gross motor skills each to provide a comprehensive picture of a child's motor repertoire (Ulrich, 2000). The TGMD is easily

Downloaded by Univ Southern Qld on 02/28/17, Volume 0, Article Number 0

© 2017 Human Kinetics, Inc.

administered and provides information on qualitative aspects and sequences of movement behaviors (Burton & Miller, 1998; Cools, De Martealaer, Samaey, & Andries, 2008; Wiart & Darrah, 2001). Valuable information may be derived from standardized observations of these FMS, which may be an important component of assessing the motor development of young children. The utilization of both process- and product-oriented approaches for the TGMD allows for ample information on movement behaviors to be derived from the assessment, which may allow practitioners, teachers, and researchers to garner more information.

A norm-referenced assessment compares performance to that of a representative, normative group, while a criterion-referenced test compares a child's performance to a set standard of performance (American Education Research Association [AERA], American Psychological Association [APA], & National Council on Measurement in Education [NCME], 2014). The TGMD is comprised of both criterion-referenced and norm-referenced components (Burton & Miller, 1998; Cools et al., 2008; Ulrich, 2000). For the normative data set used with the TGMD, the representative sample is updated every 15 years to reflect changes in the population from the United States census data (AERA, APA, & NCME, 2014; ProQuest, 2012). The TGMD strives to include FMS information in the normative data set that is representative of children between the ages of 3 to 10 years of age in the United States. Therefore, the data that is compiled must be reflective of the composition of age, sex, ethnicity/race, geographic region, and socioeconomic status based on the most recent United States census data (Kaplan & Saccuzzo, 2009; ProQuest, 2012). During this revision of the normative data set, feedback is compiled from experts in the field, researchers, and practitioners who use the test to ensure these FMS are still relevant and appropriate for the targeted purposes. With this revision for the TGMD-3 comes the necessary

Downloaded by Univ Southern Qld on 02/28/17, Volume 0, Article Number 0

© 2017 Human Kinetics, Inc.

evaluation of the psychometric properties of this assessment to ensure that usage is both valid and reliable.

Validity, or the degree to which a measurement adequately assesses constructs of interest, is a critical component of test creation and revision (AERA, AOA, & NCME, 2014; Urbina, 2004). An important underlying element of establishing validity is that the purpose of the assessment needs to match the intended interpretation. Like the TGMD-2, the purposes of the TGMD-3 include: 1) identification and screening; 2) instructional programming; 3) assessment of an individual's progress; 4) program evaluation; and 5) research tool, in relation to FMS competency in young children (Ulrich, 2000). The conceptual framework for the TGMD-3 focuses on evaluating a well-representative variety of FMS in young children, and has been supported through previous editions to align with the intended purposes (Ulrich, 1985, 2000). Validation work for a subsample of the TGMD-3 normative data (i.e., item analysis sample) will be discussed in this paper and reinforce this framework as changes have been made to the TGMD-3. New validation and reliability measures must be examined to ensure this test is appropriate prior to expanding data collection to fit the current census information of the U.S. population and finalizing the normative data set (AERA, APA, & NCME, 2014).

Reliability, or the consistency of performance across multiple occasions, is imperative to ensure an assessment is free from random error measurements (AERA, APA, & NCME, 2014; Urbina, 2004). Reliability of a measure provides an estimate of the average amount of error in a child's test score. This can be particularly relevant when decisions of services or program evaluations may hinge on assessments being reliable (Kaplan & Saccuzzo, 2009). Based on the stated purposes of the TGMD-3, it is important to establish reliability prior to evaluating the various forms of validity. Assessment literature highlights that a tool may not be valid if it is proven

Downloaded by Univ Southern Qld on 02/28/17, Volume 0, Article Number 0

© 2017 Human Kinetics, Inc.

Downloaded by Univ Southern Qld on 02/28/17, Volume 0, Article Number 0

to be unreliable (AERA, APA, & NCME, 2014; Kaplan & Saccuzzo, 2009; Urbina, 2004). Therefore, the purpose of this manuscript is to investigate the reliability and validity of the TGMD-3. Specifically, to ensure that an initial subsample of children provide acceptable forms of validity and reliability so that the normative data set may be established.

## Methods

### *Participants*

In this investigation of the psychometric properties of the TGMD-3, 807 children between 3-10.9 years of age (*M* age = 6.33 ± 2.09 years) were included in the analysis. This group of participants will be included in the full normative data set that will be published in the manual of the TGMD-3 (Ulrich, 2016). Of the participants, 52.5% were male and the racial make-up for this group included: 57.3% Caucasian, 18.0% African American, 13.3% Hispanic, 7.4% Asian/ Pacific Islander, 0.2% American Indian/ Eskimo/ Aleut, and 3.8% mixed racial background. Participants were recruited through elementary schools and preschools across the United States; University of Michigan Institutional Review Board approval was gained as well as parental consent and child assent prior to data collection.

Test-retest reliability was also evaluated for a subsample of children from this group (*n* = 30; 60% male). Children were randomly selected to participate in this process.

### *Test of Gross Motor Development – 3rd edition*

The TGMD-3 is a direct observation assessment that measures performance of 13 FMS. These skills are partitioned into two subscales, locomotor and ball skills. The skills assessed in the locomotor subscale include: run, gallop, one-legged hop, skip, jump, and slide. The ball skills evaluated include: two-hand strike, one-hand strike, catch, kick, dribble, overhand throw, and

© 2017 Human Kinetics, Inc.

underhand throw. Each skill is evaluated by examining three to five performance criteria. For example, the performance criteria for skipping include: 1) *A step forward followed by a hop on the same foot*; 2) *Arms are flexed and move in opposition to legs to produce force*; and 3) *Completes four continuous rhythmical alternating skips*.

For each skill, a trained administrator demonstrates the skill and then provides the participant one practice trial, followed by two formal trials that will be observed by the administrator and coded. Each skill has three to five performance criteria for each movement pattern. If a child correctly demonstrated the performance criteria, they were awarded a score of "1" for each trial. If they do not demonstrate the appropriate criteria, a score of "0" was recorded for the trial. Total scores from the performance criteria over the two formal trials are summed to create a raw skill score. Skill scores may be summed to provide a total raw score for either the locomotor or ball skills subscales, or combined to provide a total TGMD-3 raw score. The locomotor subscale raw score total has a possibility of 46 points; the ball skills subscale has a possibility of 54 points. The higher the score, the better the performance on the assessment.

Administrators were trained by the author of the assessment and reached 98% reliability coding sample administrations prior to testing. The author of the assessment and a motor development specialist were the primary administrators for this particular component of the project. One administrator conducted the assessment while another coded the assessments live, or video recorded them for later analysis. Children were tested individually or in small groups of two to four participants. All video recorded assessments (approximately 50% of the assessments) were coded by at least two administrators, 98% inter-rater reliability was achieved for these reliability checks. All live coded assessments were coded by the two main administrators (i.e., test author

Downloaded by Univ Southern Qld on 02/28/17, Volume 0, Article Number 0

© 2017 Human Kinetics, Inc.

Downloaded by Univ Southern Qld on 02/28/17, Volume 0, Article Number 0

and motor development specialist). To ensure 98% of agreement was being met, approximately 50% of the live observations were coded simultaneously by these two administrators.

For the children that were randomly selected to participate in the test-retest protocol, the same administrator who conducted the first assessment with the child administered the TGMD-3 a second time. Children were retested at least one week after their first administration, the amount of time varied ($M$ = 13.23 days, range = 7 – 25 days) depending on the school's schedule, holidays, or absences. Average age of children from this group was 6.33 years (range: 3 – 10 years).

*Statistical Analysis*

For each statistical test, raw scores were used and calculated for the locomotor and ball skills subscales, as well as for the total TGMD-3. Descriptive statistics were calculated and subdivided by age group (i.e., 1 group for each year between 3 to 10 years). Additionally, correlations were calculated between average performance for each subscale and age. Internal consistency measures were evaluated using Cronbach's coefficient alphas for each age group as well as several subgroups (i.e., both sexes and different ethnic/racial groups). Reliability coefficients that are greater than or equal to 0.70 are considered minimally reliable while reliability coefficients greater than or equal to 0.90 are considered ideal (Nunnally & Bernstein, 1994). Test-retest reliability was assessed on a subsample of children ($n$ = 30) using the intra-class correlation coefficient (ICC). ICC agreements were rated as poor for values less than 0.40, fair to good for values ranging from 0.40 - 0.75, and excellent if they were above 0.75 (Nunnally & Bernstein, 1994).

Item difficulty was calculated by examining proportions of success; acceptable cutoffs were deemed acceptable in the 15-85% range (Anastasi & Urbina, 1997). Item discrimination was

© 2017 Human Kinetics, Inc.

calculated by the item-total-score Pearson correlation index, cutoff of indexes .20 or higher was considered minimally acceptable (Anastasi & Urbina, 1997).

The overall sample was randomly split in two (subsample one, $n$ = 407; subsample two, $n$ = 400). Exploratory factor analysis (EFA) with maximum likelihood estimation procedures was used to examine the factor structure of the TGMD-3 on subsample one. Eigenvalues of one or greater (Kaiser, 1974) and a scree test were used as factor extraction criteria. The chi-square ($\chi^2$) value, factor loadings (> .40) and cross-loadings (< .30), and communalities were used to guide model and parameter fit (Brown, 2015). These criteria, collectively, were used to make comparisons between plausible solutions (i.e., one-factor model; two-factor model).

A confirmatory factor analysis (CFA) with maximum likelihood estimation procedures was used to validate construct validity findings from the EFA. Specifically, the factor structure produced in the retained EFA on subsample one was confirmed with the second subsample. However, we also tested an alternative model representing the EFA solution that was not retained for comparison purposes. Both parameters and model fit indexes were used to judge the adequacy of the CFA. Standardized factor loadings of .40 or greater were again used as minimum parameter criteria. The overall $\chi^2$ value provided an assessment of the absolute fit of the model with lower values (e.g., non-significant $p$-values) representing a good fit. However, $\chi^2$ tests are considered highly conservative and restrictive tests that rarely produce non-significant $p$-values (Kline, 2016). Therefore, CFA model evaluation was also determined by the following criteria (Brown, 2015; Kline, 2016): (a) comparative fit index (CFI) close to .90 - .94 = acceptable fit, .95+ = good fit; (b) Tucker-Lewis Index (TLI) close to .90 - .94 = acceptable fit, .95+ = good fit; (c) root mean square error of approximation (RMSEA) close to .08 = acceptable fit, .06 = good fit; and (d) standardized root mean square residual (SRMR) close to .08 = acceptable fit, .06 = good fit. The

Downloaded by Univ Southern Qld on 02/28/17, Volume 0, Article Number 0

© 2017 Human Kinetics, Inc.

Statistical Package for Social Sciences version 23.0 (SPSS Inc., Chicago, IL, USA) was used for all calculations except CFAs (lavaan package in R); alphas levels were set *a priori* to 0.05.

**Results**

The ball skills subscale had a slightly higher average raw score compared to the locomotor subscale, 38.2 ($SD$ = 14.9) and 33.0 ($SD$ = 12.0), respectively. As previously denoted, a higher raw score indicates higher motor skill competency and the raw scores improve with increasing age over the eight age groups (Table 1). Also, moderate to large correlations were found between average raw scores for each category and age (Cohen, 1988), with ball skills having a higher correlation ($r$ = 0.47, moderate correlation) compared to locomotor skills ($r$ = 0.39, moderate correlation; Table 1).

Internal consistency was very high in each age group for both subscales and total raw scores (Table 2). Cronbach's coefficient alpha levels all exceeded the 0.90 excellent classification with the exception of one group, which was ball skills for 10-year-old children ($\alpha$ = 0.89; Cronbach, 1951). When examining the internal consistency for various subgroups (gender and ethnicity), values remained at an excellent level; however, the American Indian/ Eskimo/ Aleut subgroup did not have a large enough sample size to calculate internal consistency (Table 2).

For test-retest reliability, ICC was determined between scores for the first and second administration. All subscales had excellent ICC agreements, the locomotor subscale had an ICC of 0.97, the ball skills subscale had an ICC of 0.95, and the total TGMD-3 scores had an ICC of 0.97.

Median item difficulty levels from the locomotor subscale had difficulty values ranging from 0.49-0.87, the ball skills subscale ranges from 0.43-0.91 (Table 3). Average values were 0.74

Downloaded by Univ Southern Qld on 02/28/17, Volume 0, Article Number 0

© 2017 Human Kinetics, Inc.

Downloaded by Univ Southern Qld on 02/28/17, Volume 0, Article Number 0

and 0.71 for locomotor and ball skills respectively for the TGMD-3, this is comparable to the average values of the TGMD-2 which were 0.77 and 0.69 for the same subscales (Table 3).

The TGMD-3 had above acceptable item discrimination values, ranging from 0.34 to 0.67, and is similar in variation to that of the TGMD-2, which ranged from 0.38 to 0.58 (Ulrich, 2000; Table 4). The average median item discrimination values were 0.52 for locomotor skills and 0.54 for ball skills; Anastasi & Urbina (1997) indicated indices greater than 0.35 are considered appropriate.

The data was randomly split into two sets to run EFA and CFA. Age, sex, and ethnicity were similar between the two subsets. For the exploratory subset (i.e., subsample one) the sample contained 407 participants, 52.1% male and average age of 6.16 years ($SD$ = 2.12 years). Using maximum likelihood EFA, one factor emerged from the data, $\chi^2$ (65) = 520.25, $p < .001$, explaining 73.82% of the variance. Examination of the scree plot clearly identifies a single factor with an eigenvalue of 9.596, well above the recommended eigenvalue of 1.0 (Kaiser, 1974). This lends to the notion that each of the 13 skills contribute to a one-factor solution, with the latent variable being gross motor skills. Factor loadings ranged from 0.797 (slide) to 0.897 (kick; $M$ = 0.85, $SD$ = 0.03), details can be found in Table 5. Communalities were high; indicating ample variance for each skill was explained by this single latent factor, ranging from 0.635 (slide) to 0.810 (kick; Table 5).

A two-factor EFA solution was also tested and although it produced a smaller chi-square value, $\chi^2$ (53) = 338.78, $p < .001$ than the one-factor model, all other criteria failed to support the two-factor solution (see Table 5). The eigenvalue of the second factor was .56. Similarly, factor one accounted for the primary factor loadings in all 13 skill indicators while the second factor did not explain meaningful variance providing clear support for the one-factor model.

© 2017 Human Kinetics, Inc.

Downloaded by Univ Southern Qld on 02/28/17, Volume 0, Article Number 0

Results from the one-factor CFA in the second subsample produced results that supported the solution identified in the one-factor EFA on subsample one. Standardized factor loadings (*M* = .84, *SD* = .05) for the 13 TGMD-3 skills ranged from 0.76 (slide) to 0.92 (kick) and were all highly significant ($p < .001$). Subsequently, communalities were also high (*M* = .71, *SD* = .08) suggesting high amounts of common variance between the single gross motor skill factor and each test. Model-related information was generally good, $\chi^2$ (65) = 327.61, $p < .001$, CFI = .95, TLI = .94, RMSEA = .10, SRMR = .03. Although the RMSEA value is higher than anticipated, the general parameter and model fit tests, collectively, confirm the construct validity of the TGMD-3 as a battery of tests representing an overall factor of gross motor development.

An alternative two-factor CFA model produced a slightly better fit compared to the one-factor CFA, $\chi^2$ (64) = 273.72, $p < .001$, CFI = .96, TLI = .95, RMSEA = .09, SRMR = .03. However, examination of parameters revealed that the correlation between the two latent factors of locomotor skills and ball skills was .96 and failed to support two clear factors. Based on all of the evidence from these EFAs and CFAs, it was concluded that the one-factor solution garnered the most empirical support and was therefore retained.

## Discussion

Assessment of the psychometric properties of a revised test is critical to ensure that the latest edition of the assessment is valid and reliable so that it may be used with confidence. Previous work has indicated that both the TGMD and TGMD-2 were suitable assessments for examining FMS in young children (Burton & Miller, 1998; Cools et al., 2008; Ulrich, 1985, 2000). From the evaluation of this initial item analysis for the TGMD-3, preliminary results show that the assessment exhibits high levels of validity and reliability. This provides confidence for the usage of this revised edition of the TGMD and supporting the collection of new norms for the TGMD-3.

© 2017 Human Kinetics, Inc.

Downloaded by Univ Southern Qld on 02/28/17, Volume 0, Article Number 0

Examining the raw scores for the TGMD-3, the ball skills subscale had a slightly higher average raw score compared to the locomotor subscale, likely due to the ball skills subscale having eight additional performance criteria compared to the locomotor subscale (Ulrich, 2016). The raw scores improved with increasing age over the eight age groups, indicating there is a developmental nature to this assessment and provides evidence for construct validity (Urbina, 2004; Table 1). Construct validity was additionally reinforced with the moderate to large correlations found between raw scores and age (Anastasi & Urbina, 1997).

Internal consistency was evaluated to examine the reliability of the assessment to ensure the TGMD-3 is homogeneous in the variables assessed (Kaplan & Saccuzzo, 2009). Reliability coefficients were found to be ideal, with the exception of one group (Nunnally & Bernstein, 1994). However, this value was minimally below the ideal mark and still achieved acceptable levels (Table 2). Internal consistency was also examined for subgroups to ensure this assessment is appropriate for both sexes and different ethnic/racial groups (AERA, APA, & NCME, 2014). Coefficients remained at an excellent level, indicating this assessment consistently evaluates closely related constructs across all ages, in both sexes as well as different ethnic/racial groups. There was also little variation among subgroups in terms of values for internal consistency, indicating its appropriateness among a wide variety of groups within this item analysis sample, further providing evidence for reliability and construct validity (Urbina, 2004). However, the American Indian/ Eskimo/ Aleut subgroup did not have a large enough sample size to calculate internal consistency, further work when completing the normative data set is needed to ensure a representative sample is acquired for this group and psychometric properties are assessed appropriately.

© 2017 Human Kinetics, Inc.

Downloaded by Univ Southern Qld on 02/28/17, Volume 0, Article Number 0

For test-retest reliability, a subsample of children were randomly selected to be assessed on two occasions to determine if the assessment adequately represented stability over time (AERA, APA, & NCME, 2014). ICC values were all in excellent agreement with one another, indicating that this assessment shows strong stability and reliability across time points (Nunnally & Bernstein, 1994). Based on the evaluation of internal consistency and test-retest reliability of this assessment, the TGMD-3 boasts a high degree of reliability. The magnitude of these results provides ample evidence of this version being used with confidence among test users in terms of this being an appropriate, reliable version of this assessment.

Item difficulty and item discrimination were used to examine content validity for this assessment (Anastasi & Urbina, 1997). For item difficulty, using the dichotomous nature of the TGMD-3, a proportion was assessed to evaluate successful achievement of all performance criteria (Urbina, 2004). Difficulty was found to reduce as children age, indicating the developmental nature of FMS that children tend to be more competent at older ages. There may be a chance that as children reach an item difficulty over 0.85 (e.g., 8 and 9 year old locomotor skills, 9 and 10 year old ball skills) that they have reached a difficulty threshold established by Anastasi and Urbina (1997) and after which may encounter a ceiling effect in terms of being competent in FMS and transitioning into more context-specific skills. The average item difficulty for the locomotor subscale was slightly higher compared to the ball skills. As this evaluation takes into account proportions of successfully completing performance criteria, this may provide more detailed information about differences in raw scores between the two subscales. Performance is also comparable to the average values from the TGMD-2 (Ulrich, 2000; Table 3).

Item discrimination, which examines how performance on one item from an assessment is related to performance on other related items, was assessed to examine content and item validity

© 2017 Human Kinetics, Inc.

Downloaded by Univ Southern Qld on 02/28/17, Volume 0, Article Number 0

(Urbina, 2004). These values are able to examine those children that performed well on certain skills should then perform similarly to related items on the assessment (Anastasi & Urbina, 1997). The TGMD-3 had above acceptable item discrimination values and is similar in variation to that of the TGMD-2 (Ulrich, 2000; Table 4). Assessments may be considered less valid if too many items contain low indexes (AERA, APA, & NCME, 2014); therefore confidence may be expanded upon from this evaluation.

Finally, EFA was examined to determine the factor structure of the latent variables underlying the TGMD-3 and to examine factor loadings for each skill on the appropriate structure. Unlike the previous editions of this assessment that have generated two factors (Ulrich, 1985; Ulrich 2000), the TGMD-3 data set had one latent variable emerge indicative of gross motor skill, with approximately 74% of variance explained (Urbina, 2004). This was further supported by CFA highlighting an appropriate fit for the one-factor model for the TGMD-3 with all skills contributing substantially. A two-factor model was tested with an EFA, as is theoretically postulated for the TGMD, however, the second factor's eigenvalue was extremely low, contributed little variance, and most cross loadings were significant to a singular factor. Also, a CFA was conducted with the split sample to test the two-factor model. The composite tests of the CFA did produce a decent fit, but due to a strong correlation between the two factors, the one factor model demonstrated a better fit for the TGMD-3 data.

When comparing the TGMD-3 and TGMD-2, the addition of three new skills and removal of two, factor loadings appear to higher for the TGMD-3 compared to the TGMD-2. The primary difference is that the TGMD-2 split into a two-factor model, for locomotor and object control skills (Ulrich, 2000). The TGMD-3 appears to strongly support a one-factor model, gross motor skill (i.e., a combination of locomotor and ball skills) which features strong variance explained and high

© 2017 Human Kinetics, Inc.

Downloaded by Univ Southern Qld on 02/28/17, Volume 0, Article Number 0

amounts of common variance shared for every skill when cross loading were removed during the CFA. This difference highlights the importance of both locomotor and object control subscales to contribute to overall gross motor skill development.

The reliability and validity measures culminated to form a body to support the intended usage and interpretation of the TGMD-3. According to the *Standards for Educational and Psychological Testing*, a portfolio of psychometric evaluations are needed to provide a comprehensive look into validity, that it is not encompassed in a singular measure (AERA, APA, & NCME, 2014). With this in mind, the validity for the item analysis portion of TGMD-3 is adequate and acceptable for usage to evaluate FMS competency.

FMS are an essential component to participation and future engagement in physical activity behaviors (Logan et al., 2015; Robinson et al., 2015). These are not naturally developing movements, rather they need to be learned and practiced for children to gain competency (Clark, 1994; Logan, Robinson, Wilson, & Lucas, 2011). Longitudinal work has found evidence that motor skill competency at a young age may be related to physical activity participation in adolescence (Barnett, van Beurden, Morgan, Brooks, & Beard, 2009) and middle adulthood (Lloyd, Saunders, Bremer, & Tremblay, 2014). Further work has found that simply targeting physical activity may not be adequate to produce long-term changes in physical activity behaviors in children without increasing FMS competency (Bryant, James, Birch, & Duncan, 2014). These studies emphasize the critical need for increasing FMS competency in young children and for valid and reliable assessments in evaluating these skills effectively. Based on the evaluations of the psychometric properties evaluated in this item analysis sample, adequate evidence has been found to support that the TGMD-3 is a valid and reliable tool for FMS assessment. The final normative

© 2017 Human Kinetics, Inc.

Downloaded by Univ Southern Qld on 02/28/17, Volume 0, Article Number 0

data set has adequate evidence to include all skills and performance criteria established in this new addition.

It is important to acknowledge the limitations of this current study. First, this is an assessment of a portion of children that will be included in the normative sample for the TGMD-3. Therefore, while census data drove recruitment for this assessment no modifications have been made at this point to ensure that the population will be representative of United States census data until the normative data collection is complete. Initial evaluation is necessary in a substantial sample to ensure the revisions are adequate and appropriate in terms of reliability and validity. Future work once the normative data set is complete to confirm these psychometric properties is needed. This includes collecting a larger group for test-retest reliability to bolster the evidence in this area, based on the large age range examined by the TGMD-3. Additionally further work on establishing a plethora of statistical evidence to provide information on the reliability and validity on the TGMD-3 is needed once the normative set is completed. This may include further investigation into inter-rater and intra-rater reliability, criterion-prediction validity with an established FMS assessment, and clinical validity for children with disabilities with the TGMD-3.

## Acknowledgments

The authors would like to sincerely thank Dr. Alex Garn (Louisiana State University, Baton Rouge) for his statistical expertise and assistance in the analysis of the statistical procedures presented here. Additionally, we would like to thank the administrators, schools, and children that participated in the collection of this data.

"Evaluation of the Psychometric Properties of the Test of Gross Motor Development – 3rd Edition"
by Webster EK, Ulrich DA
*Journal of Motor Learning and Development*
© 2017 Human Kinetics, Inc.

## References

American Education Research Association, American Psychological Association, & National Council on Measurement in Education (2014). *Standards for Educational and Psychological Testing.* Washington, DC: American Educational Research Association.

Anastasi, A., & Urbina, S. (1997). *Psychological testing* (7th ed.). Upper Saddle River, NJ: Prentice-Hall.

Barnett, L.M., van Beurden, E., Morgan, P.J., Brooks, L.O., & Beard, J.R. (2009). Childhood motor skill proficiency as a predictor of adolescent physical activity. *Journal of Adolescent Health, 44*, 252-259.

Brown, T. A. (2015). *Confirmatory factor analysis for applied research* (2nd ed.). New York, NY: Guilford Press.

Bryant, E.S., James, R.S., Birch, S.L., & Duncan, M. (2014). Prediction of habitual physical activity level and weight status from fundamental movement skill level. *Journal of Sport Sciences, 32*(19), 1775-1782.

Burton, A.W., & Miller, D.E. (1998). *Movement skill assessment.* Champaign, IL: Human Kinetics.

Clark, J.E. (1994). Motor development. In V.S. Ramachandran (Ed.), *Encyclopedia of human behavior* (Vol. 3, pp. 245-255). San Diego, CA: Academic Press.

Clark, J.E., & Metcalfe, J.S. (2002). The mountain of motor development: A metaphor. In J.E. Clark & J.H. Humphrey (Eds.), *Motor development: Research and Reviews* (Vol. 2, pp. 163–190). Reston, VA: National Association of Sport and Physical Education.

Cohen, J. (1988). *Statistical Power Analysis for the Behavioural Sciences*, 2nd ed. Hillsdale, NJ: Erlbaum.

Cools, W., De Martealaer, K., Samaey, C., & Andries, C. (2008). Movement skill assessment of typically developing preschool children: A review of seven movement skill assessment tools. *Journal of Sports Science and Medicine, 8,* 154-168.

Cronbach, L.J. (1951). Coefficient alpha and the internal structure of tests. *Psychometrika, 16*, 297-334.

Haywood, K. M., & Getchell, N. (2014). *Lifespan motor development* (6th ed.). Champaign, IL: Human Kinetics.

Kaiser, H.F. (1974). Little Jiffy, Mark IV. *Educational and Psychological Measurement, 34,* 111-117.

Kaplan, R. M., & Saccuzzo, D. P. (2009). *Psychological testing: Principles, applications, and issues* (7th ed.). Belmont, CA: Wadsworth Cengage Learning.

Downloaded by Univ Southern Qld on 02/28/17, Volume 0, Article Number 0

Kline, R. B. (2016). *Principles and practice of structural equation modeling* (4th ed.). New York, NY: Guilford Press.

Logan, S.W., Robinson, L.E., Wilson, A.E., & Lucas, W.A. (2012). Getting the fundamental of movement: A meta-analysis of the effectiveness of motor skill interventions in children. *Child: Care, Health, and Development, 38*(3), 305-315.

Logan, S.W., Webster, E.K., Getchell, N., Pfieffer, K.A., & Robinson, L.E. (2015). Relationship between fundamental motor skill competence and physical activity during childhood and adolescence: A systematic review. *Kinesiology Reviews, 4*(4), 416-426.

Lloyd, M., Saunders, T.J., Bremer, E., & Tremblay, M.S. (2014). Long-term importance of fundamental motor skills: A 20-year follow-up study. *Adapted Physical Activity Quarterly, 31*, 67-78.

Malina, R. M., Bouchard, C., & Bar-Or, O. (2004). *Growth, maturation, and physical activity* (2nd ed.). Champaign, IL: Human Kinetics.

Nunnally, J.C., & Bernstein, I.H. (1994). *Psychometric theory* (3rd ed.). New York City, NY: McGraw-Hill.

Piek, J.P., Dawson, L., Smith, L.M., & Gasson, N. (2008). The role of early fine and gross motor development on later motor and cognitive ability. *Human Movement Science, 27*, 668-681.

ProQuest LLC. (2012). *ProQuest statistical abstracts of the United States, 2013* (1st ed.). Bethesda, MD: Bernan.

Robinson, L.E., Stodden, D.F., Barnett, L.M., Lopes, V.P., Logan, S.W., Rodrigues, L.P., & D'Hondt, E. (2015). Motor competence and its effect on positive development trajectories of health. *Sports Medicine, 45*(9), 1273-1284.

Stodden, D.F., Goodway, J.D., Langendorfer, S.J., Roberton, M.A., Rudisill, M.E., Garcia, C., & Garcia, L.E. (2008). A developmental perspective on the role of motor skill competence in physical activity: An emergent relationship. *Quest, 60*, 290-306.

Ulrich, D.A. (1985). *Test of Gross Motor Development.* Austin, TX: Pro-Ed.

Ulrich, D.A. (2000). *Test of Gross Motor Development* (2nd ed.). Austin, TX: Pro-Ed.

Ulrich, D.A. (2016). *Test of Gross Motor Development* (3rd ed.). Austin, TX: Pro-Ed.

Urbina, S. (2004). *Essentials of psychological testing.* Hoboken, NJ: John Wiley & Sons, Inc.

Wiart, L., & Darrah, J. (2001). Review of four tests of gross motor development. *Developmental Medicine & Child Neurology 43*, 279-285.

Downloaded by Univ Southern Qld on 02/28/17, Volume 0, Article Number 0

"Evaluation of the Psychometric Properties of the Test of Gross Motor Development – 3rd Edition"
by Webster EK, Ulrich DA
*Journal of Motor Learning and Development*
© 2017 Human Kinetics, Inc.

## Table 1. Raw Score Means (and Standard Deviations) by Age for TGMD-3 Subtests

| Age | *n* | Locomotor | Ball Skills | Total TGMD-3 |
|---|---|---|---|---|
| 3 | 72 | 23.3 (14.2) | 25.0 (16.8) | 48.3 (30.5) |
| 4 | 131 | 26.4 (13.7) | 28.6 (16.8) | 55.0 (30.1) |
| 5 | 116 | 31.2 (12.1) | 34.8 (15.3) | 66.0 (26.7) |
| 6 | 125 | 35.3 (9.8) | 40.7 (12.5) | 76.0 (21.5) |
| 7 | 110 | 35.4 (9.4) | 42.1 (10.4) | 77.4 (18.7) |
| 8 | 104 | 37.0 (9.1) | 43.7 (10.2) | 80.7 (18.4) |
| 9 | 81 | 37.7 (8.9) | 45.2 (9.6) | 83.0 (17.6) |
| 10 | 68 | 38.7 (7.7) | 48.4 (6.2) | 87.1 (13.0) |
| Average | | 33.0 (12.0) | 38.2 (14.9) | 71.1 (26.2) |
| Correlation with age | | 0.391** | 0.474** | 0.448** |
| **Subgroups** | ***n*** | **Locomotor** | **Ball Skills** | **Total TGMD-3** |
| Male | 424 | 32.7(12.2) | 39.4(14.3) | 72.1(25.8) |
| Female | 383 | 33.2(11.8) | 36.8(15.4) | 70.0(26.6) |
| Caucasian | 462 | 35.0(11.1) | 40.6(13.6) | 75.6(24.1) |
| African American | 145 | 28.1(10.7) | 32.7(14.4) | 60.8(24.2) |
| Hispanic | 107 | 32.7(14.7) | 38.9(17.0) | 71.6(31.2) |
| Asian/ Pacific Islander | 60 | 27.7(11.9) | 29.9(16.0) | 57.6(26.7) |
| Two or more | 31 | 36.0(10.7) | 41.7(12.6) | 77.6(23.2) |

** Correlation is significant at the 0.01 level (2-tailed).

*****American Indian/ Eskimo/ Aleut was not calculated due to low sample size

Downloaded by Univ Southern Qld on 02/28/17, Volume 0, Article Number 0

“Evaluation of the Psychometric Properties of the Test of Gross Motor Development – 3rd Edition”
by Webster EK, Ulrich DA
*Journal of Motor Learning and Development*
© 2017 Human Kinetics, Inc.

## Table 2. Internal consistency for the TGMD-3, Cronbach alpha’s

| | Subtest | | |
|---|---|---|---|
| **Age Groups** | **Locomotor** | **Ball Skills** | **Total TGMD-3** |
| 3 | 0.97 | 0.97 | 0.98 |
| 4 | 0.97 | 0.97 | 0.98 |
| 5 | 0.96 | 0.97 | 0.98 |
| 6 | 0.94 | 0.96 | 0.97 |
| 7 | 0.94 | 0.93 | 0.96 |
| 8 | 0.94 | 0.94 | 0.97 |
| 9 | 0.94 | 0.94 | 0.97 |
| 10 | 0.93 | 0.89 | 0.95 |
| Mean | 0.95 | 0.95 | 0.97 |
| **Subgroups** | | | |
| Male | 0.96 | 0.97 | 0.98 |
| Female | 0.96 | 0.97 | 0.98 |
| Caucasian | 0.96 | 0.97 | 0.98 |
| African American | 0.95 | 0.96 | 0.97 |
| Hispanic | 0.98 | 0.98 | 0.99 |
| Asian/ Pacific Islander | 0.95 | 0.97 | 0.98 |
| Two or more | 0.95 | 0.97 | 098 |
| Overall | 0.92 | 0.95 | 0.96 |

*****American Indian/ Eskimo/ Aleut was not calculated due to low sample size

Downloaded by Univ Southern Qld on 02/28/17, Volume 0, Article Number 0

"Evaluation of the Psychometric Properties of the Test of Gross Motor Development – 3rd Edition"
by Webster EK, Ulrich DA
*Journal of Motor Learning and Development*
© 2017 Human Kinetics, Inc.

**Table 3. Median Item Difficulties for TGMD-3 Scores at Eight Age Intervals**

| Age | TGMD-3 | | Total TGMD-3 | TGMD-2 | |
|---|---|---|---|---|---|
| | Locomotor | Ball Skills | | Locomotor | Ball Skills |
| 3 | 0.49 | 0.43 | .47 | 0.36 | 0.32 |
| 4 | 0.56 | 0.52 | .53 | 0.60 | 0.46 |
| 5 | 0.70 | 0.62 | .63 | 0.75 | 0.59 |
| 6 | 0.80 | 0.74 | .76 | 0.86 | 0.71 |
| 7 | 0.81 | 0.79 | .79 | 0.87 | 0.79 |
| 8 | 0.86 | 0.82 | .83 | 0.92 | 0.83 |
| 9 | 0.85 | 0.86 | .86 | 0.91 | 0.88 |
| 10 | 0.87 | 0.91 | .90 | 0.91 | 0.90 |
| Avg | 0.74 | 0.71 | 0.72 | 0.77 | 0.69 |

**Table 4. Median Discriminating Powers for TGMD-3 Scores at Eight Age Intervals**

| Age | TGMD-3 | | Total TGMD-3 | TGMD-2 | |
|---|---|---|---|---|---|
| | Locomotor | Ball Skills | | Locomotor | Ball Skills |
| 3 | 0.65 | 0.67 | 0.65 | 0.58 | 0.46 |
| 4 | 0.62 | 0.67 | 0.64 | 0.56 | 0.48 |
| 5 | 0.59 | 0.62 | 0.60 | 0.58 | 0.48 |
| 6 | 0.44 | 0.57 | 0.53 | 0.46 | 0.40 |
| 7 | 0.43 | 0.46 | 0.44 | 0.39 | 0.39 |
| 8 | 0.49 | 0.46 | 0.46 | 0.38 | 0.42 |
| 9 | 0.50 | 0.51 | 0.49 | 0.41 | 0.42 |
| 10 | 0.45 | 0.34 | 0.40 | 0.43 | 0.44 |
| Avg | 0.52 | 0.54 | 0.53 | 0.47 | 0.44 |

Downloaded by Univ Southern Qld on 02/28/17, Volume 0, Article Number 0

“Evaluation of the Psychometric Properties of the Test of Gross Motor Development – 3rd Edition”
by Webster EK, Ulrich DA
*Journal of Motor Learning and Development*
© 2017 Human Kinetics, Inc.

## Table 5. Maximum likelihood exploratory factor analysis for TGMD-3

| | One-Factor Model | | Two-Factor Model | | |
|---|---|---|---|---|---|
| | Factor 1 | | Factor 1 | Factor 2 | |
| Skill Indicators | *λ* | *h²* | *λ* | *λ* | *h²* |
| Run | .82 | .67 | .82 | .02 | .66 |
| Gallop | .82 | .68 | .77 | -.22 | .71 |
| Hop | .89 | .80 | .83 | -.36 | .93 |
| Skip | .84 | .71 | .80 | -.21 | .74 |
| Jump | .85 | .71 | .80 | -.22 | .75 |
| Slide | .80 | .64 | .78 | -.05 | .63 |
| Two-hand strike | .82 | .67 | .87 | .19 | .72 |
| One-hand strike | .87 | .76 | .89 | .08 | .77 |
| Dribble | .87 | .76 | .89 | .07 | .77 |
| Catch | .85 | .72 | .87 | .09 | .73 |
| Kick | .90 | .81 | .90 | .01 | .80 |
| Overhand throw | .87 | .76 | .90 | .10 | .79 |
| Underhand throw | .80 | .64 | .83 | .11 | .66 |
| Eigenvalue | 9.59 | | 9.59 | 0.56 | |
| % Explained Variance | 73.82 | | 73.82 | 4.27 | |

Note. $\lambda$ = standardized factor loading; $h^2$ = communality.

Downloaded by Univ Southern Qld on 02/28/17, Volume 0, Article Number 0
