# Supplementary material for: Associations between fundamental movement skills and accelerometer-measured physical activity in Chinese children: the mediating role of cardiorespiratory fitness
Source: PeerJ. 2024 Jun 24;12:e17564. doi: 10.7717/peerj.17564 (PMC11210481; doi:10.7717/peerj.17564)
Supplement: Supplemental Information 3 [file peerj-12-17564-s003.pdf]

ResearchGate

See discussions, stats, and author profiles for this publication at: https://www.researchgate.net/publication/308391469

International variability in 20 m shuttle run performance in children and youth: Who are the fittest from a 50-country comparison? A systematic literature review with pooling of ag...

Article *in* British Journal of Sports Medicine · September 2016

DOI: 10.1136/bjsports-2016-096224

CITATIONS
114

READS
3,305

5 authors, including:

Justin J. Lang
Public Health Agency of Canada
163 PUBLICATIONS **20,308** CITATIONS

SEE PROFILE

Luc Leger
Université de Montréal
193 PUBLICATIONS **9,518** CITATIONS

SEE PROFILE

Tim Olds
University of South Australia
432 PUBLICATIONS **27,529** CITATIONS

SEE PROFILE

Grant Tomkinson
University of South Australia
214 PUBLICATIONS **7,302** CITATIONS

SEE PROFILE

All content following this page was uploaded by Tim Olds on 08 October 2019.

The user has requested enhancement of the downloaded file.

International variability in 20 m shuttle run performance in children and youth: who are the fittest from a 50-country comparison? A systematic literature review with pooling of aggregate results

Justin J Lang,[1] Mark S Tremblay,[1] Luc Léger,[2] Tim Olds,[3] Grant R Tomkinson[3,4]

▸ Additional material is published online only. To view please visit the journal online (http://dx.doi.org/10.1136/bjsports-2016-096224).

[1]Healthy Active Living and Obesity (HALO) Research Group, Children's Hospital of Eastern Ontario Research Institute, Ottawa, Ontario, Canada
[2]Département de kinésiologie, Université de Montréal, Montreal, Quebec, Canada
[3]Alliance for Research in Exercise, Nutrition and Activity (ARENA), School of Health Sciences & Sansom Institute for Health Research, University of South Australia, Adelaide, South Australia, Australia
[4]Department of Kinesiology and Public Health Education, University of North Dakota, Grand Forks, North Dakota, USA

Correspondence to
Justin J Lang, Healthy Active Living and Obesity Research Group, Children's Hospital of Eastern Ontario Research Institute, 401 Smyth Rd, Ottawa, Ontario, Canada K1H 8L1; jlang@cheo.on.ca

Accepted 29 August 2016

To cite: Lang JJ, Tremblay MS, Léger L, *et al*. *Br J Sports Med* Published Online First: [*please include* Day Month Year] doi:10.1136/bjsports-2016-096224

ABSTRACT

Objectives To describe and compare 20 m shuttle run test (20mSRT) performance among children and youth across 50 countries; to explore broad socioeconomic indicators that correlate with 20mSRT performance in children and youth across countries and to evaluate the utility of the 20mSRT as an international population health indicator for children and youth.

Methods A systematic review was undertaken to identify papers that explicitly reported descriptive 20mSRT (with 1-min stages) data on apparently healthy 9–17 year-olds. Descriptive data were standardised to running speed (km/h) at the last completed stage. Country-specific 20mSRT performance indices were calculated as population-weighted mean z-scores relative to all children of the same age and sex from all countries. Countries were categorised into developed and developing groups based on the Human Development Index, and a correlational analysis was performed to describe the association between country-specific performance indices and broad socioeconomic indicators using Spearman's rank correlation coefficient.

Results Performance indices were calculated for 50 countries using collated data on 1 142 026 children and youth aged 9–17 years. The best performing countries were from Africa and Central-Northern Europe. Countries from South America were consistently among the worst performing countries. Country-specific income inequality (Gini index) was a strong negative correlate of the performance index across all 50 countries.

Conclusions The pattern of variability in the performance index broadly supports the theory of a physical activity transition and income inequality as the strongest structural determinant of health in children and youth. This simple and cost-effective assessment would be a powerful tool for international population health surveillance.

Background

Cardiorespiratory endurance (CRE) is described as a trait that is partly determined by genetic factors[1] and modifiable by physical activity.[2] There is growing evidence of an inverse relationship between CRE and a variety of important health markers in children and youth.[3–6] Being a trait, CRE is less labile than physical activity,[7] and thus tracks moderately well from childhood to adulthood.[8–10] In addition, longitudinal studies have identified a significant graded association between low CRE in late adolescence and an increase in the risk of cardiovascular disease[11] and early death[12] in adulthood. Taken together, these findings indicate that the population status of CRE in children and youth may help predict future non-communicable disease burden. As a result, there have been recent calls in North America,[13 14] South America,[15] Europe[16] and Oceania[17] to monitor CRE in children and youth through continuous surveillance efforts.

The 20 m shuttle run test (20mSRT)[18 19] is considered the best and most popular field-based measurement of CRE in children and youth because of its low cost, simplicity and ability to test large groups of children simultaneously.[20–22] In 9–17 years, it demonstrates moderate-to-high criterion-related validity, indicating that it is a good surrogate measure of maximal oxygen uptake,[23] the underlying construct of CRE. Novel data standardisation techniques also allow for meaningful international comparisons of 20mSRT performance in youth.[24] Recent studies have identified large variability in children's 20mSRT performance within and between countries,[6 15 25–27] which may be associated with broad population-level socioeconomic, cultural and policy factors (henceforth referred to as socioeconomic indicators). Consequently, as a surveillance instrument, the 20mSRT could help identify populations with high CRE and provide insights into broad socioeconomic indicators that may inform intervention, programme and/or policy targets to help improve CRE in other populations, especially those with low CRE.

In 2006, Olds *et al*[27] published a seminal study describing the worldwide variability in 20mSRT performance in children and youth across 37 countries. That study represented an effort to describe and rank the international state of CRE in children and youth, serving as a comparison standard for countries. As a result, some countries were motivated to assess and verify their international 20mSRT performance rank[28] and others to establish country-specific normative data to determine their international rank compared to the standard.[17 29] However, since 2006 it is possible that the variability in 20mSRT performance has changed due to temporal trends in children's CRE that have been reported to differ in magnitude and direction at the country level.[22 24] These findings support the need for a 10-year update of Olds *et al*'s initial study.

Thus, the objectives of this paper were threefold: (1) to update, describe and compare the 20mSRT

performance of children and youth across countries; (2) to explore broad, country-level, socioeconomic indicators that correlate with 20mSRT performance in children and youth and (3) to evaluate the utility of the 20mSRT as an international population health indicator for children and youth.

METHODS

Data source

A systematic review of literature was conducted to locate studies that reported descriptive 20mSRT data on apparently healthy (free from known disease/injury) 9–17 year-olds. The search strategy for this study is reported in detail by Tomkinson *et al*.[30] Briefly, researchers conducted a search of five databases, including MEDLINE (1946–2015), PsycINFO (1806–2014), EMBASE (1947–2014), SPORTDiscus (1949–2014) and Cochrane Central Register of Controlled Trials (2005–2014). The search strategy included the following terms: shuttle run*, OR beep test, OR multi-stage, OR aerobic, OR cardio*, OR endurance; with child*, adolescen*, pubescen* boy, girl, young and youth as search term modifiers. No search restriction was placed on language. Studies were excluded if they reported data on special interest groups (eg, elite athletes and mentally/physically disabled) or unhealthy/injured groups. Consequently, studies were included if participants were broadly representative of their source population and if descriptive data were provided for 20mSRT performance (using the 1-min stage protocol) at the sex by age by country level. The reference lists for all included studies were reviewed for additional manuscripts not identified through the database search. When needed, attempts were made to contact study authors to clarify study details, to request raw data and/or to ask if they knew of other relevant studies. In addition, the personal libraries of the study authors were reviewed for relevant studies.

Inclusion/exclusion

A total of 979 studies were identified as relevant. Of these, 802 studies were excluded for one or more of the following reasons: (1) an unknown version of the 20mSRT; (2) descriptive data were combined for girls and boys into a single group; (3) study participants were atypical of the general population (eg, elite athletes or physically delayed); (4) the study reported duplicate data already reported in another included study and (5) sample size at each sex by age by study group was <40 participants as the means and SDs for smaller samples were too labile. All 20mSRT performance data from the remaining 177 studies were extracted into Excel (Microsoft Office 2010, USA) using a standardised template (see online supplementary material 1 for a description of the studies).

Standardisation of data and statistical analysis

The goal of data standardisation was to express all 20mSRT performance scores as a common protocol and result metric to facilitate cross-country comparisons. Figure 1 describes the general methodological approach used in this study. In brief, three common protocol variants for the 20mSRT were identified,[24] with all performances standardised to the Léger protocol.[19] This protocol begins at a speed of 8.5 km/h for the first stage (min) and increases by 0.5 km/h every stage thereafter. Furthermore, 20mSRT performances were reported in one of the six different result metrics (speed at the last completed stage, completed stage or minutes, distance, completed laps within final stage, total laps or estimated $VO_{2\,peak}$) with all results standardised to running speed (km/h) at the last completed stage. Details on data standardisation procedures are described by Tomkinson *et al*.[24 30]

The main objective of this study was to compare 20mSRT performances across countries. To achieve this, sample-weighted mean and SD 20mSRT performance values were calculated for each sex by age by country group and used to calculate sex- by age- by country-specific mean z-scores using the international 20mSRT performance norms (table 1) reported by Tomkinson *et al*.[30]

$$\text{Mean z-score} = \sum \frac{\bar{X} - \mu}{\sigma}$$

where $\bar{X}$ is the country sample-weighted mean sex- by age-specific performance score, μ is the international mean performance score for the given sex- by age-specific group and σ is the international SD for the given sex- by age-specific group (see table 1).

Positive z-scores indicated that 20mSRT performances were better than the international mean and negative z-scores that

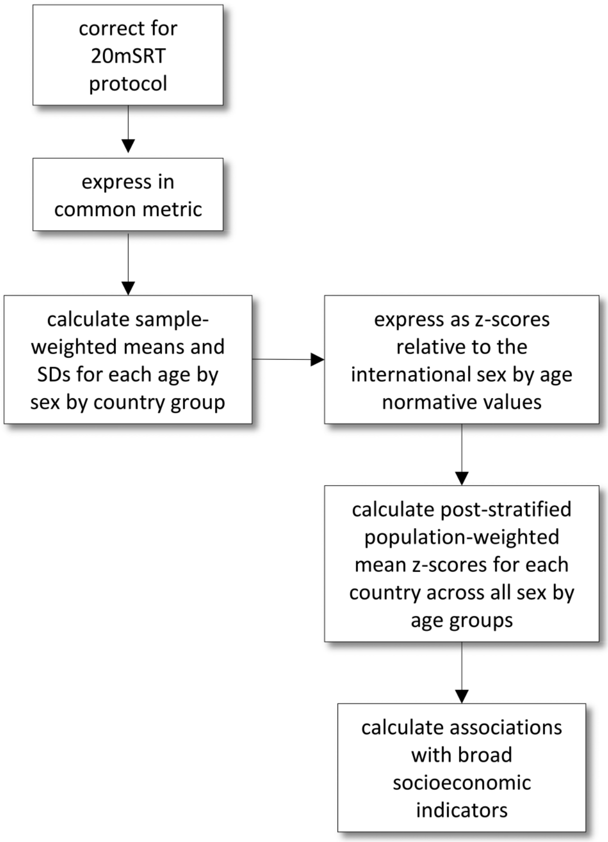

Figure 1 Flow chart describing the general methodological procedure for this study. All protocol results (Léger, Eurofit or Queen's University of Belfast) were corrected to reflect the Léger protocol. All results were then corrected to reflect speed (km/h) at the last completed stage as a common result metric. Next, sample-weighted mean 20mSRT performance score and SD were calculated for each sex by age by country group. Then z-scores were generated relative to the international norms for each sex by age by country group to calculate the mean country specific z-score, described as the 'performance index.' Each country specific performance index was corrected using a poststratified population-weighted technique. Finally, a correlation analysis took place to analyse associations between the performance index and broad socioeconomic indicators. 20mSRT, 20 m shuttle run test.

Table 1 International 20mSRT norms for running speed (km/h) at the last completed stage[30]

	Boys			Girls		
Age	N	Mean	SD	N	Mean	SD
9	101 532	10.03	0.85	98 166	9.71	0.70
10	81 538	10.13	0.92	81 415	9.75	0.77
11	79 660	10.25	1.00	78 029	9.79	0.83
12	70 266	10.47	1.11	69 497	9.83	0.88
13	72 913	10.73	1.21	62 491	9.86	0.91
14	65 317	10.97	1.28	55 035	9.89	0.93
15	53 287	11.12	1.33	43 730	9.91	0.94
16	35 490	11.27	1.37	34 978	9.93	0.95
17	29 123	11.41	1.42	29 559	9.96	0.97
Total	589 126			552 900		

20mSRT, 20 m shuttle run test.

20mSRT performances were worse than the international mean. Population-weighted mean z-scores and corresponding 95% CIs were calculated for each country, across all sex by age groups for which data were available, using a poststratification population-weighting procedure described by Levy and Lemeshow.[31] Population estimates standardised to the mean measurement year of 2000 were extracted from the United Nations World Population Prospective report, that's what we mean by mean measurement year.[32] These population-weighted z-scores represented the country-specific 'performance index', that is, the overall standardised deviation of a country's children 20mSRT performance from the international age- and sex-specific means. The index served as a method to rank and compare the overall performance of each country.

The secondary objective was to explore correlates between the 20mSRT performance index and broad socioeconomic indicators. An internet search of publicly available databases was conducted to identify socioeconomic indicators that, based on the literature, would be meaningful to explore. In total, six indicators were identified (table 2), and data were extracted at the country level. The association between the 20mSRT performance index and each socioeconomic indicator was quantified using Spearman's rank correlation coefficient with the 95% confidence limits calculated using Fisher's z-transformation. Holm–Bonferroni sequential correction was used to adjust for α slippage.[39] Correlations of 0.1, 0.3 and 0.5 were used as thresholds for weak, moderate and strong, respectively, with correlations <0.1 considered to be trivial.[40] In addition, countries were grouped by the human development index (HDI) into developed (HDI of 0.741–0.943) and developing (HDI of 0.286–0.740) nations using the 2011 inequality-adjusted United Nations Human Development Report.[36] The association between the 20mSRT performance index and each indicator was further explored by HDI status to identify potential interactions. Combined, the first two objectives of this study helped inform and evaluate the utility of the 20mSRT as an international population health indicator for children and youth.

RESULTS

Cross-country variability

Standardised 20mSRT performance indices are presented across 50 countries, representing 1 142 026 children and youth aged 9–17 years. The 50 countries represented six of the seven continents (North and South America, Europe, Africa, Asia and Oceania), and a wide range of HDI (0.400 (Côte d'Ivoire) to 0.943

Table 2 Potential correlates of 20mSRT performance in children and youth

Variable	Data source	Description
Childhood obesity (%)	Ng *et al*[33]<br>Data available for 49 countries	Calculated as the mean country-specific proportion of boys and girls aged <20 years that classified as obese based on the International Obesity Task Force definition. Countries with a higher proportion received a higher rank.
Physical activity (%)	Kalman *et al*[34]<br>Data originally obtained from HSBC study<br>Data available for 22 developed countries	Calculated as the mean country-specific percentage of boys and girls aged 11, 13 and 15 years that achieved at least 1 hour of moderate-to-vigorous physical activity per day. Countries with a higher proportion received a higher rank.
Mean climate (°C)	Weatherbase[35]<br>Data available for 50 countries	Calculated across the number of years and cities where data were available (ie, Canada had 26 years of data across 2287 cities). Colder countries received a higher rank.
HDI	The United Nations[36]<br>Data available for 50 countries	Summarises country-specific achievement in a variety of indicators related to standards of living through a scale from 0.456 (low HDI) to 0.889 (very high HDI). High HDI received a higher rank.
Gini index	The World Bank[37]<br>Data available for 48 countries	Summarises the distribution of wealth among individuals in a country where 0 represents perfect equality and 100 implies perfect inequality. Countries closest to inequality received a higher rank.
Urbanisation (%)	The World Bank[38]<br>Data available for 46 countries	Proportion of the population that reside in an urban area within the country. Countries with a higher percentage of urban areas received a higher rank.

20mSRT, 20 m shuttle run test; HDI, human development index.

(Norway)).[36] The performance index values for each of the 50 countries are presented in table 3 along with their respective 95% CI and centile rank. Confidence in our performance index scores varied substantially between countries based on country-specific sample size. Performance indices ranged from +1.85 for Tanzania (the best performing country) to −0.96 for Mexico (the worst performing country). Countries from Central and Northern Europe and Africa accounted for 9 of the top 10 performing countries. South American countries were consistently among the worst performing countries, with all countries except Colombia and Suriname, falling below the international mean (mean±95% CI: centile 40±11; z-score −0.27±0.32). Collectively, Africa (mean ±95% CI: centile 68±8; z-score 0.53±0.32) was typically the best performing continent, closely beating Europe (mean±95% CI: centile 63±7; zscore 0.37±0.21). However, when dividing Europe into two segments, similar to that performed by Ortega *et al*,[193] a performance gradient existed where Central-Northern European countries (mean±95% CI: centile 66±7; z-score 0.48 ±0.23) consistently outperformed countries from Southern Europe (mean±95% CI: centile 45±7; z-score −0.12±0.19).

Correlates of 20mSRT performance

The Gini index was a moderate to strong negative correlate of 20mSRT performance, regardless of country development status (table 4). Other correlations across the sample ranged from

Table 3 Descriptive data for countries presented in rank order of by 2016 performance index

Country	N	Performance index±95% CI	Centile rank
Tanzania[41]	274	1.85±0.08	97
Iceland[42]	6130	1.45±0.03	93
Estonia[43–48]	8013	1.42±0.02	92
Norway[29]	2305	1.15±0.04	87
Japan[49]	279 732	0.98±0.01	84
Denmark[50]	9342	0.85±0.03	80
Czech Republic[51 52]	1953	0.75±0.05	77
Finland[51 53 54]	5679	0.74±0.03	77
Côte d'Ivoire[55]	3273	0.68±0.04	75
Morocco[56]	2522	0.65±0.03	74
France[16 57–62](G Cazorla, JP Colin, L Léger. Condition physique des jeunes collégiens et lycéens de la région aquitaine (France). Unpublished manuscript, 1999).	7244	0.59±0.03	72
Lithuania[43 45]	6479	0.52±0.02	70
Benin[63]	4141	0.51±0.03	70
Djibouti[64]	1524	0.48±0.04	68
Mauritius[65]	2695	0.47±0.03	68
Slovakia[66–71]	4827	0.39±0.03	65
Germany[16 51]	1146	0.38±0.06	65
Sénégal[72]	2243	0.37±0.04	64
Canada[18 73–77]	29 009	0.32±0.01	63
Turkey[78]	45	0.32±0.20	63
UK[79–91] (RM Ranson, S Taylor. Unpublished 'F' Factor Report for Denbighshire County Council. Wrexham, UK: Glyndwr University, 2010)	49 140	0.31±0.01	62
Poland[92–95]	58 681	0.30±0.01	62
Suriname[96]	270	0.30±0.08	62
Belgium[16 51 97–105]	23 358	0.28±0.01	61
China[106]	548	0.25±0.07	60
Sweden[16]	45	0.20±0.37	58
Austria[16]	70	0.19±0.23	58
Netherlands[107–109]	3457	0.18±0.03	57
Spain[16 110–122]	25 950	0.14±0.01	56
South Africa[123–130]	2970	0.09±0.04	54
Switzerland[131 132]	3224	0.08±0.03	53
Uganda[133]	1446	0.08±0.05	53
Seychelles[134 135]	9174	0.07±0.02	53
Colombia[136–138]	36 117	0.05±0.01	52
Australia[139–149] (S Mulkearns, G Naughton, J Carlson, et al. The effects of additional physical education on health-related fitness measures of circumpubertal females. Unpublished manuscript, 2000.)	26 719	−0.01±0.01	50
Hungary[16 51 150 151]	3294	−0.08±0.04	47
Italy[16 152–157]	5900	−0.09±0.02	46
Hong Kong[158]	1214	−0.11±0.05	46
Chile[15 159]	17 057	−0.13±0.01	45
Argentina[160]	1259	−0.16±0.05	44
Philippines[161]	340	−0.19±0.11	42
Greece[51 162–166]	268 382	−0.26±0.00	40
Portugal[167–172] (Oliveira personal communication)	30 764	−0.26±0.01	40
Brazil[173–175]	4528	−0.34±0.03	37
Cyprus[176 177]	6902	−0.34±0.03	37
Republic of Korea[178]	82	−0.38±0.12	35
USA[179–189]	171 058	−0.47±0.01	32
Latvia[190]	3400	−0.64±0.03	26
Peru[191]	560	−0.67±0.06	25
Mexico[192] (Esteve-Lanao personal communication)	7541	−0.96±0.02	17

Continued

Table 3 Continued

trivial to moderate. Mean climate was a strong positive correlate for only developed countries. In contrast, HDI was a strong negative correlate for only developing countries. Furthermore, a potential interaction existed where some socioeconomic indicators were positively correlated with 20mSRT performance in developed countries, but negatively associated in developing countries.

DISCUSSION

This study represents the largest international surveillance effort to describe the status of CRE in children and youth by presenting standardised 20mSRT performance indices across 50 countries. The aim of the present study was to update, describe and compare 20mSRT performance in children and youth across countries; to explore associations between 20mSRT performance and broad country-level socioeconomic indicators and to assess the utility of the 20mSRT as an international population health indicator for children and youth.

International variability in 20mSRT performance

There was a very high correlation (r=0.90) between the 33 common country performance indices reported in this study and in Olds *et al.*[27] Furthermore, our results suggest that 20mSRT performances in children and youth vary substantially across countries, spanning 2.8 SDs from the best performing country (Tanzania) to the worst performing country (Mexico). To illustrate this finding, figure 2 depicts the country-specific mean distance (m) run on the 20mSRT by boys and girls, which indicates that boys and girls from Tanzania would cover a distance approximately four times greater than their Mexican counterparts (equivalent to a maximal running speed of ∼1.3 times faster). The magnitude of variability in CRE between countries is concerning and suggests that low CRE may be a public health issue in some countries. Indeed, this substantial variability provides some evidence supporting CRE as a population-level indicator of health in children and youth. Future research should further explore the direct impact of low CRE in childhood and the future disease outcomes in adulthood. However, following a quartile framework, countries that fall below the 25th centile should take action by implementing policies and programmes targeted at increasing CRE in children and youth. Likewise, countries that fall between the 25th and 50th centile should take precautionary measures.

A total of 23 European countries are represented in this study. Our findings suggest a clear latitudinal gradient where Central-Northern countries perform better on the 20mSRT than their Southern counterparts by ∼0.6 SDs. Following the same Central-Northern versus Southern division of Europe, Ortega *et al*[193] also found a difference in CRE where Central-Northern countries performed significantly better on the 20mSRT than

Table 4 Spearman's rank correlation coefficients (95% CI) between 20mSRT performance index and socioeconomic indicators

	All countries (n=50)	Developed (n=34)	Developing (n=16)
Childhood obesity (%)	−0.19 (−0.44 to 0.09)	−0.27 (−0.56 to 0.08)	−0.00 (−0.50 to 0.50)
Physical activity (%)		−0.12 (−0.52 to 0.32)	
Mean climate (C°)	0.26 (−0.02 to 0.50)	0.63** (0.37 to 0.80)	−0.21 (−0.64 to 0.32)
HDI	0.01 (−0.27 to 0.29)	0.29 (−0.05 to 0.57)	−0.56 (−0.83 to −0.09)
Gini index	−0.44* (−0.64 to −0.18)	−0.70** (−0.84 to −0.47)	−0.49 (−0.79 to 0.01)
Urbanisation (%)	−0.01 (−0.30 to 0.28)	0.15 (−0.20 to 0.47)	−0.45 (−0.80 to 0.13)

Developed=high and very high (0.741–0.943) HDI; developing=very low, low and medium (0.286–0.740) HDI.
* indicates significant result at p<0.05; ** indicates significant result at p<0.01; adjusted using Holm–Bonferroni sequential correction.
20mSRT, 20 m shuttle run test; HDI, Human development index.

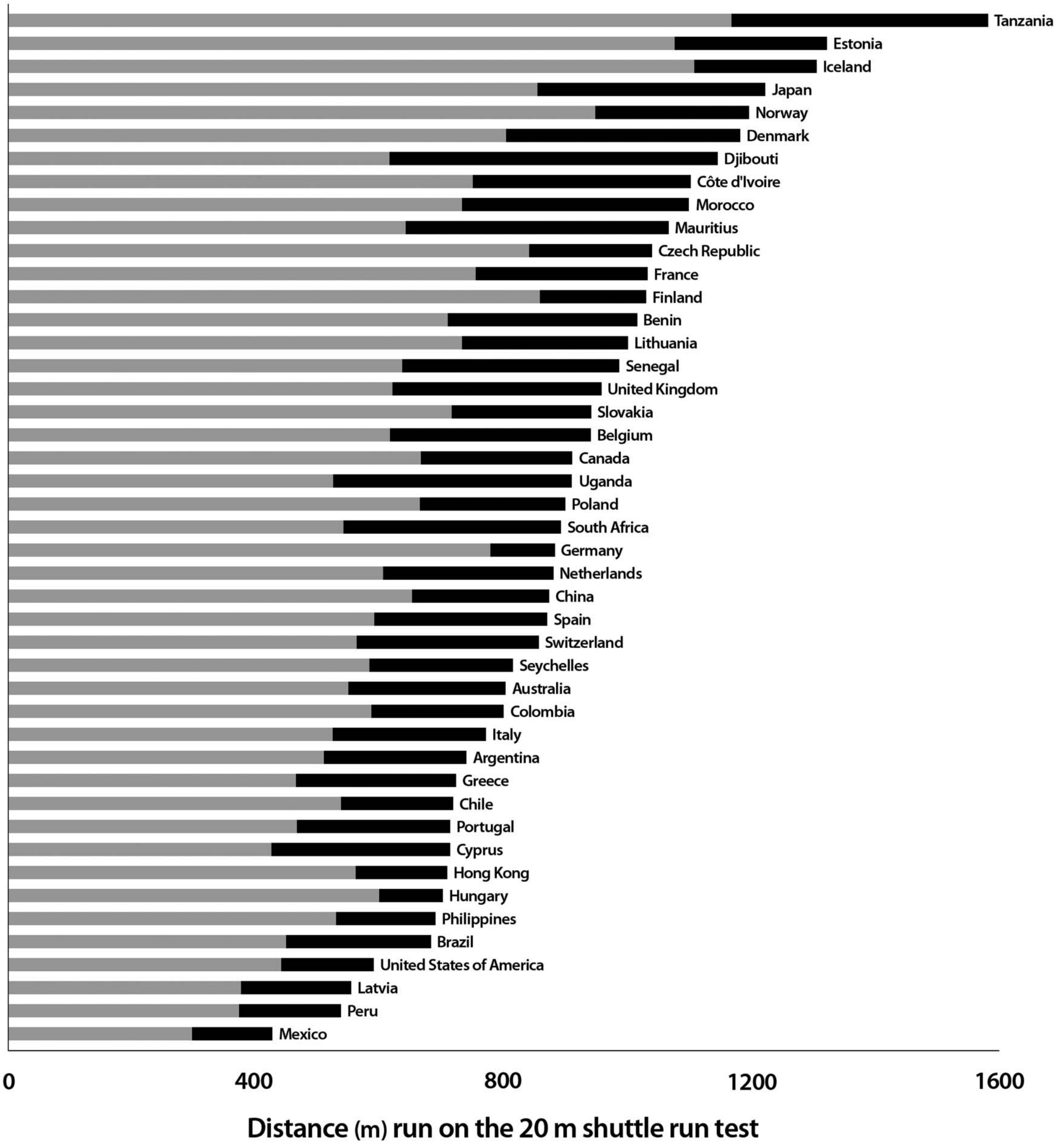

Figure 2 Bar graph describing the distance (m) run by boys and girls on the 20mSRT based on country- specific and age-specific performance indices. Only countries (n=45) with 20mSRT performance indices for boys (represented by the black bars) and girls (represented by the grey bars) are shown. 20mSRT, 20 m shuttle run test.

their Southern counterparts. These findings have been attributed to differences in physical activity levels in children and youth across countries,[194 195] which may be further exacerbated by disparities in socioeconomic indicators.[196]

Within countries we found sex differences, where boys consistently outperformed girls on the 20mSRT across all countries (figure 2). Sex differences in CRE are consistently reported in the literature,[27 197] with our study providing a unique

perspective that suggests the magnitude of the sex differences may be country specific. These sex-related differences may be due to age-related and maturation-related differences between countries. Further analysis of cross-country sex differences is beyond the scope of this paper. Nevertheless, there is a need for further research on this topic.

Possible socioeconomic correlates

Following an ecological framework,[198] highlighting correlates of 20mSRT performance may provide country-level target areas for policies and programmes that may ultimately help increase CRE in children and youth. There are many possible broad country-level correlates of 20mSRT performance in children and youth. These may include HDI, the distribution of wealth within a country, urbanisation, climate and country-specific physical activity and childhood obesity levels.

Childhood obesity

Internationally, the measurement and surveillance of body composition is a public health norm with prevalence data available for ~188 countries.[33] Studies indicate that, similar to CRE, the prevalence of overweight and obesity in children and youth varies substantially across countries.[33] Our study supports childhood obesity prevalence as a weak negative correlate of 20mSRT performance in developed countries (r_s=−0.27; 95% CI: −0.56 to 0.08). In developing countries, the correlation between childhood obesity prevalence and 20mSRT performance was trivial (r_s=−0.00; 95% CI: −0.50 to 0.50), suggesting that childhood obesity prevalence is a stronger negative correlate of CRE in more developed countries.

Physical activity

Physical activity is considered a modifier of CRE. Thus, we would anticipate that higher population levels of physical activity result in higher CRE levels in children and youth.[199] However, physical activity is a behaviour that varies substantially across time and is difficult to measure at the population level.[7 200 201] Validated self-report surveys are the most popular way to estimate physical activity across countries in children and youth, such as the Global School-based Student Health Survey[202] and the Health Behaviour in School-aged Children (HBSC) survey.[203] Data from the HBSC study suggest that moderate-to-vigorous physical activity is a weak negative correlate of 20mSRT performance in children and youth in developed countries (r_s=−0.12; 95% CI: −0.52 to 0.32). This result may reflect limitations of the HBSC study which used self-report measures for selected age groups across less than half of the countries for which performance index data were available. In contrary, these results may also reflect the fact that moderate-to-vigorous physical activity is not a good correlate of maximal-effort CRE in children and youth.

More recently, efforts have been made to use objective measures of physical activity (pedometers and accelerometers),[194] but these efforts have been predominantly adopted in developed countries.[204] However, a recent international study comparing objectively measured physical activity suggests that children in Northern Europe and Africa are more active than children in South America, broadly consistent with our findings.[205]

Urbanisation

Worldwide, there is a major population demographic shift where now, more than ever, the majority of the world's population lives in urban and/or suburban settings.[206] These demographic shifts are synonymous with a variety of lifestyle transitions as a result of mechanisation replacing laborious jobs, and an increase in transportation choices and food availability. A population shift towards cities is described as a component of the epidemiological[207] and physical activity[208] transitions due to the resulting decrease in habitual physical activity in these populations. The physical activity transition is a theoretical perspective that can be described as:

> 'Overall, socioeconomic and demographic changes occurring in concert with the epidemiological transition appear to be associated with the adoption of a lifestyle characterized by lower energy requirements at work and for the procurement of food, less dependence on walking as a primary means of transportation, and the popularity of sedentary activities to fill a growing availability of leisure time no longer needed to carry out activities related to securing food and shelter. (ref. 208, p. 277)'

Since physical activity is a modifier of CRE, there are plausible associations between high urbanisation and low CRE levels in children and youth.

In our study, urbanisation was a weak correlate of 20mSRT performance for developed countries (r_s=0.15; 95% CI: −0.20 to 0.47). This result is most likely due to developed countries already attaining a high level of urbanisation. In contrast, urbanisation is a moderate negative correlate of 20mSRT performance in developing countries (r_s=−0.45; 95% CI: −0.80 to 0.13). Although this finding is not statistically significant, it describes the potential negative impact of rapid urbanisation with regard to CRE. This pattern is especially apparent in some South American countries (Brazil, Peru and Colombia) with high urbanisation and low CRE levels. Therefore, these countries might be in the midst of a physical activity transition. This is a drastically different demographic situation compared to countries with relatively low urbanisation rates (Uganda, Sénégal, Mauritius and Benin) that demonstrate high CRE levels in children and youth.

Human Development Index

The HDI is a composite measure combining country-specific prosperity and various indicators of quality of life. Our study indicates that HDI is a weak positive correlate of 20mSRT performance in developed countries (r_s=0.29; 95% CI: −0.05 to 0.57). In contrast, developing countries show an opposite gradient where HDI is a strong negative correlate of 20mSRT performance in children and youth (r_s=−0.56; 95% CI: −0.83 to −0.09). A likely explanation of these results is that physical activity in developing countries is often obligatory because food is relatively scarce, and demanding chores required for survival. However, when these countries become more developed, food becomes readily available and the presence of mechanisation and automation makes physical activity no longer obligatory. In developed countries, it is likely that leisure time and wealth provides alternative opportunities for physical activity pursuits. These results lend further support to the presence of a physical activity transition in developing countries.[208]

Gini index

The Gini index is a population measure of the distribution of wealth. Our study indicates that a large population distribution in wealth (income inequality) is a moderate to strong negative correlate of CRE in children and youth, regardless of country development status. This finding suggests that policies aimed at reducing the gap between rich and poor might be a suitable population approach to increase CRE. Our results align with previous research that highlighted income inequality as the

strongest structural determinant of health in adolescents.[209] Furthermore, Elgar *et al*[210] found that high national income inequality was associated with low population levels of physical activity, high BMI and more psychological and physical health symptoms in youth.

Climate

Our study indicates that climate is a strong positive correlate of 20mSRT performance (r_s=0.63; 95% CI: 0.37 to 0.80) for developed countries, where colder countries have higher CRE than warmer countries. In developing countries, climate is a weak negative correlate (r_s=−0.21; 95% CI: −0.64 to 0.32), where warmer countries tend to perform better than their colder counterparts. These results are conflicting and difficult to interpret. It is possible that the strong positive correlation in developed countries is due to the negative physiological effect of exercising in warm and humid temperatures. However, in contrast to previous findings, the strong correlation in developed countries remained significant after removing Northern European countries from the analysis (r_s=0.53; 95% CI: 0.21 to 0.75), suggesting that the result was not an artefact of the superior performance of children and youth in Northern Europe.[27]

Strengths and limitations

Our study represents the largest and most comprehensive cross-country comparison of children's CRE. Although this study is not the first of its kind, it does provide a significant update to the study by Olds *et al*[27] by extending the coverage date from 2003 to 2015, and by adding an additional 13 countries and 724 000 data points to the analysis. As a result of the broad scope of this study, we acknowledge several limitations: (1) globally, CRE has declined by 2.2% per decade from 1981 to 2015 (for all children), with little change over the past 15 years. This decline may have artificially increased the rankings of countries with measurements that were made earlier (ie, Benin in 1987, Côte d'Ivoire in 1985, Turkey in 1991 and Suriname in 1991) and decreased those that were made more recently; (2) we combined a variety of studies with different sampling strategies (probability and non-probability) and sampling frames (national, state/provincial and regional); (3) some country-specific performance indices were calculated using small samples and limited sex by age groups which reduced the confidence in some of our country estimates; (4) the testing conditions may have varied between included studies (ie, indoor/outdoor, altitude and temperature); (5) measurement drift may have occurred in administering the 20mSRT between and within countries and (6) individual physiological (eg, mechanical efficiency,[211] fractional utilisation[212] and lactate threshold[213]), psychosocial (eg, motivation and self-efficacy[20]) and cultural factors may have also contributed to differences in performance and may have changed in some countries over time.

To strengthen our study, we combined data from 177 studies, representing the best available data on 20mSRT performance in children and youth, using rigorous and standardised data treatment techniques. These techniques control for variability in reporting 20mSRT results and provide us with the ability to compare testing results meaningfully between studies. We used a poststratification population-weighting procedure, so that our performance index reflects the country-specific population demographics. Furthermore, we believe that the size of our study, with over 1.1 million test performances, will help decrease any study specific limitations.

Surveillance of CRE as a powerful population health indicator in children and youth

Convincing evidence indicates that the precursors for future disease have their origin in childhood and adolescence, making these age groups very important for current and future health.[214] Thus, childhood and adolescent health promotion efforts in the form of interventions, programmes and policies are important public and population health initiatives. However, it is impossible to know the impact of such efforts without the consistent surveillance and monitoring of population health indicators. Currently, there are two comparable population health indicators in children and youth—body weight status and physical activity levels. The surveillance of body weight status at a population level is essential, but changes in body weight do not occur fast enough to understand the short- and medium-term impact of newly implemented policies and programmes. Evidence also suggests that individuals can classify as obese but still have healthy CRE and a healthy phenotype, known as the obesity paradox.[215] Ideally, monitoring physical activity levels in a population would provide important feedback on health promotion efforts, but there remain several limitations in the effectiveness and feasibility of measuring physical activity in populations of children and youth.[200 201] Although physical activity and CRE are independently associated with health markers,[216] conceptually, CRE is a possible proxy for overall physical activity levels within a population. Thus, the measurement of CRE, via the 20mSRT, can be an objective surrogate measure that can be used to study the link between physical activity and health. As a population health indicator, the 20mSRT is an effective and feasible tool that demonstrates minimal barriers with regard to culture and language, is cost-effective, results are easy to score and interpret, and it can be easily administered with large groups of children.[20] We believe that the population-wide and international measurement of CRE is important to understand the health of children and youth, the impact of health promotion policies and programmes as well as the potential future population burden of non-communicable disease.

Recommendation

We recommend the development of an international 20mSRT internet portal that provides resources to correctly administer and record 20mSRT performances following the recommendations of Tomkinson *et al*.[30] This easy-to-access portal would also include an international 20mSRT data repository where researchers, physical educators, coaches and individuals could input their performance data for children and youth free of charge. In return, the website would compute a results sheet comparing the group/individual performance score with international,[30] regional and country normative centile values, providing the user with general performance feedback. All data would be housed on a secure server and used to continuously update international normative values, cross-country comparisons and temporal trends in 20mSRT performance. This data repository would serve as an international surveillance system to monitor 20mSRT performance in children and youth. The authors are currently working to launch such a repository.

CONCLUSION

Our study demonstrates the broad international utility of the 20mSRT in children and youth. This field-based assessment is suitable for international surveillance because of its low cost, ease in administering, very good test-retest reliability and

validity as well as ability to test large groups of children simultaneously. This study used a systematic review method, rigorous data treatment and a poststratification population-weighting procedures to obtain and compare 20mSRT performance data across 50 countries. Our data suggest that children and youth in Central-Northern Europe and Africa are among the world's best 20mSRT performers, whereas those from South America are among the worst performers. Worldwide patterns of variability in 20mSRT performance broadly mirror socioeconomic indicators in a way that support the theory of a physical activity transition and the importance of wealth distribution as a strong structural determinant of health. These data can help inform the development of an international 20mSRT surveillance system to monitor the variability and temporal trends in 20mSRT performance for children and youth across countries, providing insights into the general health and well-being of populations.

What are the findings?

- This study provides a 50-country comparison of 20 m shuttle run performance using rigorous data standardisation techniques.
- There are broad country-specific socioeconomic indicators that correlate with 20 m shuttle run performance in children and youth.
- 20 m shuttle run test data can be used as an international population health indicator for surveillance in children and youth.

Acknowledgements The authors acknowledge all individuals who provided details of their study and/or generously offered their raw data.

Contributors JJL, GRT and MST developed the research questions and objectives. JJL and GRT led the data analysis and synthesis of results. JJL wrote the manuscript. All authors contributed to interpreting results, editing, reviewing and approved the final manuscript.

Competing interests None declared.

Provenance and peer review Not commissioned; externally peer reviewed.

REFERENCES

1. Tucker R, Collins M. What makes champions? A review of the relative contribution of genes and training to sporting success. Br J Sports Med 2012;46:555–61.
2. Gutin B, Yin Z, Humphries MC, *et al*. Relationships of moderate and vigorous physical activity to fitness and fatness in adolescents. *Am J Clin Nutr* 2005;81:746–50.
3. Ortega FB, Ruiz JR, Castillo MJ, *et al*. Physical fitness in childhood and adolescence: a powerful marker of health. *Int J Obes (Lond)* 2008;32:1–11.
4. IOM (Institute of Medicine). *Fitness measures and health outcomes in youth*. Washington DC: The National Academies Press, 2012:111–51.
5. Ruiz JR, Ortega FB, Rizzo NS, *et al*. High cardiovascular fitness is associated with low metabolic risk score in children: the European Youth Heart Study. *Pediatr Res* 2007;61:350–5.
6. dos Santos FK, Prista A, Gomes TNQF, *et al*. Body mass index, cardiorespiratory fitness and cardiometabolic risk factors in youth from Portugal and Mozambique. *Int J Obes (Lond)* 2015;39:1467–74.
7. Ridley K, Olds T, Hands B, *et al*. Intra-individual variations in children's physical activity patterns: Implications for measurement. *J Sci Med Sport* 2009;12:568–72.
8. Malina RM. Physical activity and fitness: pathways from childhood to adulthood. *Am J Hum Biol* 2001;13:162–72.
9. Ortega FB, Ruiz JR, Labayen I, *et al*. Role of socio-cultural factors on changes in fitness and adiposity in youth: a 6-year follow-up study. *Nutr Metab Cardiovasc Dis* 2013;23:883–90.
10. Ruiz JR, Castro-Piñero J, Artero EG, *et al*. Predictive validity of health-related fitness in youth: a systematic review. *Br J Sports Med* 2009;43:909–23.
11. Högström G, Nordström A, Nordström P. High aerobic fitness in late adolescence is associated with a reduced risk of myocardial infraction later in life: a nationwide cohort study in men. *Eur Heart J* 2014;35:3133–40.
12. Högström G, Nordström A, Nordström P. Aerobic fitness in late adolescence and the risk of early death: a prospective cohort study of 1.3 million Swedish men. *Int J Epidemiol*. Published Online First: 20 December 2015.
13. Kaminsky LA, Arena R, Beckie TM, *et al*. The importance of cardiorespiratory fitness in the United States: the need for a national registry: a policy statement from the American Heart Association. *Circulation* 2013;127:652–62.
14. Morrow JR Jr and Ede A. Research Quarterly for Exercise and Sport lecture. Statewide physical fitness testing: a big waist or a big waste? *Res Q Exerc Sport* 2009;80:696–701.
15. Garber MD, Sajuria M, Lobelo F. Geographic variation in health-related physical fitness and body composition among Chilean 8th graders: a nationally representative cross-sectional study. *PLoS ONE* 2014;9:e108053.
16. Ortega FB, Artero EG, Ruiz JR, *et al*. Physical fitness levels among European adolescents: the HELENA study. *Br J Sports Med* 2011;45:20–9.
17. Catley MJ, Tomkinson GR. Normative health-related fitness values for children: analysis of 85347 test results on 9-17 year-old Australians since 1985. *Br J Sports Med* 2013;47:98–108.
18. Léger L, Lambert J, Goulet A, *et al*. [Aerobic capacity of 6 to 17-year-old Quebecois—20 meter shuttle run test with 1 minute stages]. *Can J Appl Sport Sci* 1984;9:64–9.
19. Léger LA, Mercier D, Gadoury C, *et al*. The multistage 20 metre shuttle run test for aerobic fitness. *J Sports Sci* 1988;6:93–101.
20. Tomkinson GR, Olds TS. Field tests of fitness. In: Armstrong N, van Mechelen W, eds. *Paediatric exercise science and medicine*. New York, NY: Oxford University Press, 2008:109–28.
21. Castro-Piñero J, Artero EG, España-Romero V, *et al*. Criterion-related validity of field-based fitness tests in youth: a systematic review. *Br J Sports Med* 2010;44:934–43.
22. Tomkinson G, Olds T. Secular changes in pediatric aerobic fitness test performance: the global picture. *Med Sport Sci* 2007;50:46–66.
23. Mayorga-Vega D, Aguilar-Soto P, Viciana J. Criterion-related validity of the 20-m shuttle run test for estimating cardiorespiratory fitness: a meta-analysis. *J Sports Sci Med* 2015;14:536–47.
24. Tomkinson GR, Léger LA, Olds TS, *et al*. Secular trends in the performance of children and adolescents (1980-2000): an analysis of 55 studies of the 20m shuttle run test in 11 countries. *Sports Med* 2003;33:285–300.
25. Adamo KB, Sheel AW, Onywera V, *et al*. Child obesity and fitness levels among Kenyan and Canadian children from urban and rural environments: a KIDS-CAN Research Alliance Study. *Int J Pediatr Obes* 2011;6:e225–32.
26. Kalabiska I, Uvacsek M, Petrekanits M, *et al*. Comparison of running performance and prevalence of overweight and obesity in Hungarian and Ukrainian adolescents. *Acta Physiol Hung* 2010;97:393–400.
27. Olds TS, Tomkinson GR, Léger L, *et al*. Worldwide variation in the performance of children and adolescents: an analysis of 109 studies of the 20-m shuttle run test in 37 countries. *J Sports Sci* 2006;24:1025–38.
28. Sandercock G, Voss C, Gladwell V. Twenty-metre shuttle run test performance of English children aged 11–15 years in 2007: comparisons with international standards. *J Sports Sci* 2008;26:953–7.
29. Haugen T, Høigaard R, Seiler S. Normative data of BMI and physical fitness in a Norwegian sample of early adolescents. *Scand J Public Health* 2014;42:67–73.
30. Tomkinson GR, Lang JJ, Tremblay MS, *et al*. International normative 20 m shuttle run values from 1,142,026 children and youth representing 50 countries. *Br J Sports Med*. Published Online First: 20 May 2016.
31. Levy PS, Lemeshow S. Stratification random sampling: further issues. In Levy PS, Lemeshow S, eds. *Sampling of populations: methods and application*. Hoboken, NJ: John Wiley & Sons, 2008:168–75.
32. United Nations, Department of Economic and Social Affairs, Population Division. World Population Prospects: The 2012 Revision, DVD Edition. 2013.
33. Ng M, Fleming T, Robinson M, *et al*. Global, regional, and national prevalence of overweight and obesity in children and adults during 1980-2013: a systematic analysis for the Global Burden of Disease Study 2013. *Lancet* 2014;384:766–81.
34. Kalman M, Inchley J, Sigmundova D, *et al*. Secular trends in moderate-to-vigorous physical activity in 32 countries from 2002 to 2010: a cross-national perspective. *Eur J Pub Health* 2015;25(Suppl 2):37–40.
35. Weatherbase. http://www.weatherbase.com/weather/countryall.php3
36. United Nations Development Programme. Human Development Reports. 2011. http://www.undp.org/content/dam/undp/library/corporate/HDR/2011%20Global%20HDR/English/HDR_2011_EN_Complete.pdf
37. The World Bank. Gini index (World Bank estimate). http://data.worldbank.org/indicator/SI.POV.GINI/countries?display=default
38. The World Bank. Urban population (% of total). http://data.worldbank.org/indicator/SP.URB.TOTL.IN.ZS?display=default
39. Holm S. A simple sequentially rejective multiple test procedure. *Scand J Statist* 1979;6:65–70.
40. Cohen J. *Statistical power analysis for the behavioral sciences*. 2nd edn. New Jersey: Lawrence Erlbaum, 1988.

41 Aandstad A, Berntsen S, Hageberg R, *et al*. A comparison of estimated maximal oxygen uptake in 9 and 10-year-old schoolchildren in Tanzania and Norway. *Br J Sports Med* 2006;40:278–92.
42 Gunnarsson HG, Sigríksson R. *Eurofit: physical fitness of Icelandic pupils at age of 6-15 years old*. Reykjavik, Iceland, 1999.
43 Jürimäe T, Volbekiene V, Jürimäe J. Changes in Eurofit test performance of Estonian and Lithuanian children and adolescents (1992-2002). In: Tomkinson GR, Olds TS, eds. *Pediatric fitness: secular trends and geographic variability*. Basel: Karger, 2007:129–42.
44 Jürimäe T, Saar M. Self-perceived and actual indicators of motor abilities in children and adolescents. *Percet Mot Skills* 2003;97:862–6.
45 Jürimäe T, Volbekiene V. Eurofit test results in Estonian and Lithuanian 11 to 17-year-old children: a comparative study. *Eur J Phys Educ* 1998;3:178–84.
46 Raudsepp L, Jürimäe T. Relationships of physical activity and somatic characteristics with physical fitness and motor skill in prepubertal girls. *Am J Hum Biol* 1997;9:513–21.
47 Raudsepp L, Jürimäe T. Relationships between somatic variables, physical activity, fitness and fundamental motor skills in prepubertal boys. *Biol Sport* 1996;13:279–89.
48 Kull M, Jürimäe T. Using the Eurofit test battery in Estonian 16-18 years old adolescents. *Acta Comment Univ Tartu* 1994;967:49–52.
49 Ministry of Education, Culture, Sports, Science and Technology. Report book on the survey of physical fitness and athletic ability. Tokyo: Ministry of Education, Culture, Sports, Science and Technology, 1999–2015.
50 Nielsen GA, Andersen LB. The association between high blood pressure, physical fitness, and body mass index in adolescents. *Prev Med* 2003;36:229–34.
51 Telama R, Naul R, Nupponen H, *et al*. *Physical fitness, sporting lifestyles and olympic ideals: cross-cultural studies on youth sport in Europe*. Schorndorf, Germany: Verlag Karl Hofmann, 2002.
52 Gajda V. *The applications of the UNIFITTEST battery at basic schools (age groups 7-14): summary report*. Ostrava: PdF OU, 1994.
53 Palomäki S, Heikinaro-Johansson P, Huotari P. Cardiorespiratory performance and physical activity in normal weight and overweight Finnish adolescents from 2003 to 2010. *J Sports Sci* 2015;33:588–96.
54 Viljanen T, Taimela S, Kujala U. Koululaisten fyysinen aktiivisuus, kestävyyskunto ja ponnistuskorkeus. *Liikunta and Tiede* 2000;37:23–6.
55 Cazorla G, Dudal J, Dieu JL. État de développement des capacités physiques des jeunes Ivoiriens 7-18 ans et plus. Rapport pour le Ministère Ivoirien de la Jeunesse et Sports et pour le Ministère Français de la Coopération. Paris, France: Ministrère Ivoirien de la Jeunesse et Sports et pour le Ministrère Français de la Coopération, 1985.
56 Dadouchi MF, Boudiab K, Yahia J, *et al*. De la détermination du profil athlétique marocain au contenu et à l'évaluation. Ministère de l'Éducation nationale et de la jeunesse. École normale supérieure de Marrakech. 2003.
57 Baquet G, Twisk JWR, Kemper HCG, *et al*. Longitudinal follow-up of fitness during childhood: interaction with physical activity. *Am J Hum Biol* 2006;18:51–8.
58 Baquet G, Berthoin S, Gerbeaux M, *et al*. High-intensity aerobic training during a 10 week one-hour physical education cycle: effect on physical fitness of adolescents aged 11 to 16. *Int J Sports Exerc Med* 2001;22:295–300.
59 Baquet G, Berthoin S, Gerbeaux M, *et al*. Assessment of the maximal aerobic speed with the incremental running field tests in children. *Biol Sport* 1999;16:23–30.
60 Cazorla G, Portes A, James F. Opération Martinique-Eval. Centre d'Evaluation Sport Santé, Fort de France (Martinique). Rapport pour L'Inspection d'Académie de la Martinique. Fort de france, Martinique: L'Education d'Académie de la Martique, 1997.
61 Cazorla G. Batterie France-Éval: Mesures, épreuves et barêmes: Évaluation des qualités physiques des jeunes Français d'âge scolaire: 7-11 ans. Rapport pour le Secrétariat d'Etat Auprès du Premier Ministre Chargé de la Jeunesse et de Sports. Paris: Ministère de la Jeunesse et de Sports, 1987.
62 Brunet J, Van Praagh E. *Batterie experimentale de tests moteurs Eurofit: rapport d'Activitie de la région auvergne —1984–1985*. Clermont-Ferrand, France: Université de Clermont-Ferrand, 1985.
63 Cazorla G, Gouthon P, Arémou R, *et al*. État de développement des capacités physiques des jeunes béninois 8-18 ans et plus. Rapport pour le Ministère Béninois de la Jeunesse et Sports et pour le Ministère Français de la Coopération. Paris, France: Ministrère Béninois de la Jeunesse et Sports et pour le Ministrère Français de la Coopération, 1987.
64 Cazorla G, Dudal J, Lefrancq L. État de développement des capacités physiques des jeunes Djiboutiens 11-18 ans et plus. Rapport pour le Ministère Djiboutien de la Jeunesse et Sports et pour le Ministère Français de la Coopération. Paris, France: Ministrère Djiboutien de la Jeunesse et Sports et pour le Ministrère Français de la Coopération, 1986.
65 Cazorla G, Dudal J, Garinet P. État de développement des capacités physiques des jeunes mauriciens 7-18 ans et plus. Rapport pour le Ministère Mauricien de la Jeunesse et Sports et pour le Ministère Français de la Coopération, 1986.
66 Krska P, Sedlacek J, Hubinak A, *et al*. General motor fitness and somatic parameters comparison between former population and present primary school girls in Ruzomberok. In press.
67 Krska P, Sedlacek J, Hubinak A, *et al*. Physical development and general motor performance of present primary school boys population in Ruzomberok. In press.
68 Kyselovicová O. Programy ærobiku z aspektu rozvoja telesnej zdatnosti dievcat na gymnáziu. *Acta Facult Educ Phys Univers Comen* 2000;41:50–61.
69 Kasa J, Majherová M. Physical and motor development of children by Eurofit. *Stud Psychol (Bratisl)* 1997;39:270–4.
70 Moravec R. Eurofit—Physique and motor fitness of the Slovak school youth. In Moravec R, Kampmiller T, Sedlácek J. eds. *Eurofit—Physique and motor fitness of the Slovak school youth*. Bratislava: Slovak Scientific Society for Physical Education and Sports, 1996:9–22.
71 Belej M, Junger J, Mikus M. Vykonnost ziakov prijatych na stredne skoly vo vychodoslovenskom regione zistovana systemom EUROFIT. *Tel Vych Sport* 1995;5:12–15.
72 Cazorla G, Dudal J, Faye J. État de développement des capacités physiques des jeunes sénégalais 7-18 ans et plus. Rapport pour le Ministère Sénégalais de la Jeunesse et Sports et pour le Ministère Français de la Coopération. Paris, France: Ministrère Sénégalais de la Jeunesse et Sports et pour le Ministrère Français de la Coopération, 1988.
73 Veldhuizen S, Cairney J, Hay J, *et al*. Relative age effects in fitness testing in a general school sample: how relative are they? *J Sports Sci* 2015;33:109–15.
74 Voss C, Sandercock G, Higgins JW, *et al*. A cross-cultural comparison of body composition, physical fitness, and physical activity between regional samples of Canadian and English children and adolescents. *Can J Public Health* 2014;104:e245–50.
75 Leone M, Kalinova E, Comtois AS. *Global motor skill assessment from the UQAC-UQAM test battery: Canadian normative values by age and gender*. Québec: Université du Québec à Chicoutimi, 2011.
76 Reed KE, Warburton DER, Whitney CL, *et al*. Secular changes in shuttle-run performance: a 23-year retrospective comparison of 9- to 11-year-old children. *Pediatr Exerc Sci* 2006;18:364–73.
77 Massicotte D. Partial curl-ups, push-ups and multistage 20 meter shuttle run, national norms for 6 to 17 year-olds. Final report submitted to Canadian Association for Health, Physical Education and Recreation (CAHPER) and Fitness and Amateur Sport Canada. Montréal: University of Quebec at Montréal, 1990.
78 Çalis M, Ergen E, Turnagöl H, *et al*. Beden egitimi derslerinin bir ögretim yili boyunca 15-16 yas grubu ögrenciler üzerindekifizyolojik etkilerinin Eurofit test bataryasi ile izlenmesi. In 3 Ulusal Spor Bilinleri Kongresi. Ankara, Turkey: Hacettepe Universitesi, 1992:367–72.
79 Sandercock G, Ogunleye A, Voss C. Six-year changes in body mass index and cardiorespiratory fitness of English schoolchildren from an affluent area. *Int J Obes (Lond)* 2015;39:1504–7.
80 Sandercock G, Voss C, Cohen D, *et al*. Centile curves and normative values for the twenty metre shuttle-run test in English schoolchildren. *J Sports Sci* 2012;30:679–87.
81 Boddy LM, Fairclough SJ, Atkinson G, *et al*. Changes in cardiorespiratory fitness in 9- to 10.9-year-old children: SportsLinx 1998-2010. *Med Sci Sports Exerc* 2012;44:481–6.
82 Sandercock G, Voss C, McConnell D. Ten year secular declines in the cardiorespiratory fitness of affluent English children are largely independent of changes in body mass index. *Arch Dis Child* 2010;95:46–7.
83 Liverpool City Council. Liverpool sports linx project 01-03: report on the health and fitness of liverpool primary and secondary school children. Liverpool, UK: Liverpool City Council, 2003.
84 Boreham C, Twisk J, Murray L, *et al*. Fitness, fatness, and coronary heart disease risk in adolescents: the Northern Ireland Young Hearts Project. *Med Sci Sports Exerc* 2001;33:270–4.
85 Watkins DC. *Ten year trends (1990-2000) in biological and behavioural risk factors for coronary heart disease in northern Irish adolescents* [thesis]. Belfast, UK: The Queen's University of Belfast, 2001.
86 Twisk JWR, Boreham C, Cran G, *et al*. Clustering of biological risk factors for cardiovascular disease and the longitudinal relationship with lifestyle of an adolescent population: the Northern Ireland Young Hearts Project. *J Cardiovasc Risk* 1999;6:355–62.
87 Mahoney C. 20-MST and PWC_{170} validity in non-Caucasian children in the UK. *Br J Sports Med* 1992;26:45–7.
88 Mahoney CA, Boreham CAG. Eurofit in Belfast primary schools. *Scot J Phys Educ* 1991;19:1–4.
89 Riddoch C, Savage JM, Murphy N, *et al*. Long term health implications of fitness and physical activity patterns. *Arch Dis Chil* 1991;66:1426–33.
90 Nichols AK, Riddoch CJ. The development of fitness test batteries for use in higher education. *In Trends and developments in physical education: Proceedings of the VIII Commonwealth and International Conference on Sport, Physical Education, Dance, Recreation and Health*; London: E & FN Spon, 1986:378–84.
91 Lewitt MS, Baker JS, Mooney GP, *et al*. Pubertal stage and measures of adiposity in British schoolchildren. *Ann Hum Biol* 2012;39:440–7.
92 Bronikowski M, Bronikowska M. Salutogenesis as a framework for improving health resources of adolescent boys. *Scand J Public Health* 2009;37:525–31.

93 Pilicz S, Przeweda R, Dobosz J, *et al*. Punktacja sprawnosci fizycznej mlodziezy Polskiej wg miedzynarodowego testu sprawnosci fizycznej: Kryteria pomiaru wydolnosci organizmu testem Coopera. Warszawa: Akademia Wychowania Fizycznego Józefa Pilsudskiego, 2003.
94 Maciaszek J, Osinski W. Poziom sprawnosci fizycznej u chlopców i dziewczat Poznanskich w wieku 10-14 lat. *Rocz Naukowe AWF Poznaniu* 2001;50:3–17.
95 Mleczko E, Ozimek M. *Rozwój somatyczny i motoryczny mlodziezy Krakowskiej miedzy 15 a 19 rokiem zycia z uwzglednieniem czynników srodowiskowych*. Kraków: Akademia Wychowania Fizycznego, 2000.
96 Lieveld J, Vrijens J, Bouckært J. Ethnic differences in body structure and physical fitness in Surinamean boys aged 14 years. In: Clæssens AL, Lefevre J, Vanden Eynde B, eds. *World-wide variation in physical fitness*. Leuven: Katholieke Universiteit Leuven, 1993:87–92.
97 Vandendriessche JB, Vandorpe BFR, Vaeyens R, *et al*. Variation in sport participation, fitness and motor coordination with socioeconomic status among Flemish children. *Pediatr Exerc Sci* 2012;24:113–28.
98 Seghers J, Rutten C. Clustering of multiple lifestyle behaviours and its relationship with weight status and cardiorespiratory fitness in a sample of Flemish 11- to 12-year olds. *Public Health Nutr* 2010;13:1838–46.
99 Verstraete SJ, Cardon GM, De Clercq DL, *et al*. A comprehensive physical activity promotion programme at elementary school: the effects on physical activity, physical fitness and psychosocial correlates of physical activity. *Public Health Nutr* 2007;10:477–84.
100 Cardon G, De Bourdeaudhuij I, De Clercq D, *et al*. Physical fitness, physical activity, and self-reported back and neck pain in elementary schoolchildren. *Pediatr Exerc Sci* 2004;16:147–57.
101 Heyters C, Marique T. *Le baromètre de la condition physique*. Bruxelles: Ministère de la Communauté française, Direction générale du sport, 2004.
102 Baquet G, Berthoin S, Padovano C, *et al*. Effects d'un cycle de course de durée de type intermittent (court-court) sur la condition physique des adolescents. *Rev Educ Phys* 2000;40:51–60.
103 Lefèvre J, Bouckært J, Duquet W, *et al*. De barometer van de fysieke fitheid van de Vlaamse jeugd 1997. De resultaten. *Sport (Bloso Brussel)* 1998;4:16–22.
104 Beunen G, Borms J, Vrijens J, *et al*. Fysieke fitheid en sportbeoefening van de Vlaamse jeugd. Volumen 1: Fysieke fitheid van de jeugd van 6 tot 18 jaar. Brussels: Bloso, 1991.
105 Poortmans J, Vlaeminck M, Collin M, *et al*. [Indirect estimation of the maximal aerobic power of a Male and female population from Brussels aged 6 to 23 years. Comparison with a direct technic for measuring maximal oxygen consumption]. J Physiol *(Paris)* 1986;81:195–201.
106 Wang PG, Gong G, Wang SQ, *et al*. Relationship of body fat and cardiorespiratory fitness with cardiovascular risk in Chinese children. *PLoS ONE* 2011;6:e27896.
107 Brouwer SI, Stol RP, Liem ET, *et al*. The role of fitness in the association between fatness and cardiometabolic risk from children to adolescence. *Pediatr Diabetes* 2013;14:57–65.
108 Slinger J, van Breda E, Kuipers H. Aerobic fitness data for Dutch adolescents (2002-2005). *Pediatr Exerc Sci* 2009;21:10–18.
109 van Mechelen W, van Lier WH, Hlobil H, *et al*. *Eurofit: handleiding met referentieschalen voor 12- tot en met 16-jarige jongens en meisjes in Nederland*. Haarlem: Uitgeverij de Vrieseborch, 1991.
110 Gulías-González R, Martínez-Vizcaíno V, García-Prieto JC, *et al*. Excess of weight, but not underweight, is associated with poor physical fitness in children and adolescents from Castilla-La Mancha, Spain. *Eur J Pediatr* 2014;173:727–35.
111 Torrijos-Niño C, Martínez-Vizcaíino V, Pardo-Guijarro MJ, *et al*. Physical fitness, obesity, and academic achievement in schoolchildren. *J Pediatr* 2014;165:104–9.
112 Chillón P, Ortega FB, Ferrando JA, *et al*. Physical fitness in rural and urban children and adolescents from Spain. *J Sci Med Sport* 2011;14:417–23.
113 Castro-Pinero J, González-Montesinos JL, Mora J, *et al*. Percentile values for muscular strength field tests in children aged 6 to 17 years: influence of weight status. *J Strength Cond Res* 2009;23:2295–310.
114 Ortega FB, Ruiz JR, Castillo MJ, *et al*. Low level of physical fitness in Spanish adolescents. Relevance for future cardiovascular health (AVENA Study). *Rev Esp Cardiol* 2005;58:898–909.
115 García Baena J. *La condición fisica en la educación secundaria. Trabajo de investigación* [thesis]. Madrid, Spain: Universidad Nacional de Educacion a Distancia, 1999.
116 Prat JA, Casamort J, Balagué N, *et al*. *Eurofit: la batería Eurofit en Catalunya*. Barcelona: Secretaria General de l'Esport, 1998.
117 Tercedor P, Delgado-Fernandez M. Condicion fisica relacionada con la salud en escolares de 10 anos de edad de Granada. *Proceedings of the II congreso internacional sobre la enseñanza de la educación física y el deporte escolar (second international congress about teaching physical education and school sport)*; 1998.
118 Sainz RM. *La batería Eurofit en Euskadi*. Vitoria-Gasteiz: Instituto Vasco de Educación Fisica, 1996.
119 Ureña F. *Valoración y baremación de la aptitud física en el alumnado de segundo ciclo de educación secundaria obligatoria de la comunidad autonoma de Murcia. Su utilización según los postulados de la reforma* [dissertation]. Murcia, Spain: Universidad de Murcia, 1996.
120 Brito Ojeda EM, Navarro Valdivielso M, García Afonso D, *et al*. *La condición física en la población escolar de gran Canaria (10-19 años)*. Las Palmas de Gran Canaria, Spain: Excmo. Cabildo Insular de Gran Canaria, 1995.
121 Sainz RM. *Aptitudes psiquicas y fisicas: Estudio ed la aptitud fisica de los adolescentes de la provincia de Vizcaya y su relacion con la personalidad* [dissertation]. Bilboa, Spain: Universidad de Deusto, 1992.
122 Rivas FJ. Valoracion de la evolucion anthropoetrica y de las caracteristicas motrices en la poblacion escolar de un centro de E.G.,B. mediante un estudio transversal. In II Congreso Galego da Educacion Fisica e o Deporte. La Coruña: Escola Galega do Deporte de la Xunta de Galicia, 1987.
123 Pienaar C, Coetzee B, Monyeki AM. The use of anthropometric measurements and the influence of demographic factors on the prediction of VO2max in a cohort of adolescents: the PAHL study. *Ann Hum Biol* 2015;42:135–43.
124 Du Toit D, Pienaar AE, Truter L. Relationship between physical fitness and academic performance in South African children. *S Afr J Res Sport PH* 2011;33:23–35.
125 Pienaar AE, Viljoen A. Physical and motor ability, anthropometrical and growth characteristics of boys in the northwest province of South Africa: a sport talent perspective. *S Afr J Res Sport Phys Educ Recreation* 2010;32:71–93.
126 Du Preez SM. *The effect of physical activity on the body composition and health related fitness of 9 to 13-year-old boys* [thesis]. Potchefstroom, South Africa: North-West University, 2008.
127 Stadler MC. *The influence of a physical activity intervention program (PAI) on the physical fitness levels, body composition and health risk behaviour of 9 to 13-year-old girls* [dissertation]. Potchefstroom, South Africa: North-West University, 2007.
128 Du Toit L, Venter RE, Potgieter JR. The relationship between cardiorespiratory fitness, body composition and physical self-perception of adolescent girls. *J Hum Mov Stud* 2005;48:353–64.
129 Van Gent M, Malan DDJ, Pienaar AE. A comparison of the anthropometric, physical and motor growth characteristics of 12-15 year old girls in the North West province with Australian girls. *Afr J Phys Health Educ Recr Dance* 2002;8:309–20.
130 Du Randt R. The 1996 South African sport talent identification project: report prepared for the Sports Information and Science Agency. Pretoria: South African Sports Commission, 1996.
131 Schmid M, Romann M, Kriemler S, *et al*. Wie kann die Fitness von Schulkindern gemessen werden? *Sportmed Sporttraumatol* 2007;55:52–61.
132 Cauderay M, Narring F, Michaud P-A. A cross-sectional survey assessing physical fitness of 9- to 19-year-old girls and boys in Switzerland. *Pediatr Exerc Sci* 2000;12:398–412.
133 Richards J, Foster C, Townsend N. Physical fitness and mental health impact of a sport-for-development intervention in a post-conflict setting: Randomised controlled trial nested within an observational study of adolescents in Gulu, Uganda. *BMC Public Health* 2014;14:619.
134 Bovet P, Auguste R, Burdette H. Strong inverse association between physical fitness and overweight in adolescents: a large school-based survey. *Int J Behav Nutr Phys Act* 2007;4:24.
135 Cazorla G, Rousseau G, Dudal J, *et al*. Évaluation des capacités motrices de l'enfant, de l'adolescent et du jeune seychellois : 7-18 ans et plus. Rapport pour le Ministère Seychelle de l'Education et pour le Ministère Français de la Coopération. Paris, France: Ministère Seychellois de l'Education et Ministrère Français de la Coopération, 1990.
136 Cubides RC, Alarcón LGA, Galvi ARG. Diferencias en la actividad física y la condición física entre los escolares de secundaria de dos programas curriculares oficiales de Bogotá, Colombia. *Nutr Hosp* 2015;32:2228–34.
137 Fernandez-Ortega JA. Estudio transversal de las cualidades funcionales de los escolares bogotanos: Valores de potencia aeróbica, potencia muscular, velocidad de desplazamiento y velocidad de reacción, de los siete a los dieciocho años. *Rev Edu Fís Deportes* 2013;32:1151–70.
138 Mojica GT, Poveda JG, Pinilla MI, *et al*. Sobrepeso, inactividad física y baja condición física en un colegio de Bogotá, Colombia. *Arch Latinoam Nutr* 2008;58:265–73.
139 Hardy LL, King L, Espinel P, *et al*. NSW Schools Physical Activity and Nutrition Survey (SPANS) 2010: full report. Sydney: NSW Ministry of Health, 2010.
140 McIntyre S. *Trends in aerobic fitness from 1994 to 2009 in 10- and 11-year-old Australian children* [thesis]. Notre Dame, IN: University of Notre Dame, 2009.
141 Booth M, Okely AD, Denney-Wilson E, *et al*. NSW Schools Physical Activity and Nutrition Survey (SPANS) 2004: full report. Sydney: NSW Department of Health, 2006.
142 Lloyd KC, Antonas KN. Nutritional habits and fitness levels of schoolchildren. *Proceedings of the Nutrition Society of Australia—Twenty-fourth annual scientific meeting, Fremantle, WA, 3–6 December*; Adelaide, SA: Nutrition Society of Australia, 2000:138.
143 Cooley D, McNaughton L. Aerobic fitness of Tasmanian secondary school children using the 20-m shuttle run test. *Percept Mot Skills* 1999;88:188–98.
144 Booth M, Macaskill P, McLellan L, *et al*. *NSW schools fitness and physical activity survey 1997*. Sydney: NSW Department of Education and Training, 1997.

145 Okely AD, Gray T, Cotton WG. Effect of an extended stay outdoor education program on aerobic fitness. In Gray T, Hayllar B, eds. *Catalysts for change: proceedings from the 10th national outdoor education conference*. Sydney, Australia, 1997:206–10.
146 Australian Council for Health, Physical Education and Recreation. *Australian fitness education award: user's manual and curriculum ideas*. Adelaide, SA: Australian Council for Health, Physical Education and Recreation, 1996.
147 Vandongen R, Jenner DA, Thompson C, *et al*. A controlled evaluation of a fitness and nutrition intervention program on cardiovascular health in 10- to 12-year-old children. *Prev Med* 1995;24:9–22.
148 Australian Sports Commission. *Norms for sport related fitness tests in Australian students aged 12-17 years*. Belconnen, ACT: Australian Sports Commission, 1994.
149 Jenner DA, Vandongen R, Beilin L. Relationships between blood pressure and measures of dietary energy intake, physical fitness, and physical activity in Australian children aged 11-12 years. *J Epidemiol Community Health* 1992;46:108–13.
150 Welk GJ, Saint-Maurice PF, Csányi T. Health-related physical fitness in Hungarian youth: age, sex, and regional profiles. *Res Q Exerc Sport* 2015;86(Suppl 1):S45–57.
151 Barabás, A. Measurement of aerobic power by field tests. In: Coudert J, Van Praagh E, eds. *Pediatric work physiology: children and exercise XVI*. Paris: Masson, 1992:39–41.
152 Grassi GP, Turci M, Sforza C. Aerobic fitness and somatic growth in adolescents: a cross sectional investigation in a high school context. *J Sports Med Phys Fitness* 2006;46:412–18.
153 Cilia G, Bazzano C, Bellucci M, *et al*. I risultati dei test Eurofit nella scuola Matteuccii di Roma. *Alcmeone* 1998;2:16–20.
154 Cilia G, Bellucci M, Bazzano C, *et al*. Eurofit 1997: Banche dati per la scuola. *Alcmeone* 1997;3:13–32.
155 Cilia G, Bellucci M, Riva M. *Eurofit 1995*. Roma: Istituto Superiore Statale di Educazione Fisica, 1996.
156 Cilia G, Bellucci M. *Eurofit: tests Europei di attitudine fisica*. Roma: Istituto Superiore Statale di Educazione Fisica, 1993.
157 Council of Europe. *Évaluation de l'aptitude physique: Eurofit batterie expérimentale*. Rome: Council of Europe, 1986.
158 Wong TW, Yu TS, Wang XR, *et al*. Predicted maximal oxygen uptake in normal Hong Kong Chinese schoolchildren and those with respiratory diseases. *Pediatr Pulmonol* 2001;31:126–32.
159 Kain J, Uauy R, Albala, *et al*. School-based obesity prevention in Chilean primary school children: methodology and evaluation of a controlled study. *Int J Obes Relat Metab Disord* 2004;28:483–93.
160 Secchi JD, García GC, España-Romero V, *et al*. Physical fitness and future cardiovascular risk in Argentine children and adolescents: an introduction to the ALPHA test battery. *Arch Argent Pediatr* 2014;112:132–40.
161 Gonzalez-Suarez CB, Grimmer-Sommers K. The association of physical activity and physical fitness with pre-adolescent obesity: an observational study in Metromanila, Phillipines. *J Phy Act Health* 8:804–10.
162 Tambalis KD, Panagiotakos DB, Psarra G, *et al*. Physical fitness normative values for 6-18-year-old Greek boys and girls, using the empirical distribution and the lambda, mu, and sigma statistical method. *Eur J Sport Sci* 2016;16:736–46. .
163 Tambalis K, Panagiotakos D, Sidossis L. Greek children living in rural areas are heavier but fitter compared to their urban counterparts: a comparative, time-series (1997-2008) analysis. *J Rural Health* 2011;27:270–7.
164 Tokmakidis SP, Kasambalis A, Christodoulos AD. Fitness levels of Greek primary schoolchildren in relationship to overweight and obesity. *Eur J Pediatr* 2006;165:867–74.
165 Manios Y, Yiannakouris N, Papoutsakis C. Behavorial and physiological indices related to BMI in a cohort of primary schoolchildren in Greece. *Am J Hum Biol* 2004;16:639–47.
166 Georgiadis G. Evaluation of physical fitness of Greek youth aged 6-18 years [dissertation]. Athens, Greece: University of Athens, 1993.
167 Santos R, Mota J, Santos DA, *et al*. Physical fitness percentiles for Portuguese children and adolescents aged 10-18 years. *J Sports Sci* 2014;32:1510–81.
168 Coelho-Silva MJ, Ronque ERV, Cyrino ES, *et al*. Nutritional status, biological maturation and cardiorespiratory fitness in Azorean youth aged 11-15 years. *BMC Public Health* 2013;13:495.
169 Rodrigues LP, Leitao R, Lopes VP. Physical fitness predicts adiposity longitudinal changes over childhood and adolescence. *J Sci Med Sport* 2013;16:118–23.
170 Marta CC, Marinho DA, Barbosa TM. Physical fitness differences between prepubescent boys and girls. *J Strength Cond Res* 2012;26:1756–66.
171 Silva G, Aires L, Mota J, *et al*. Normative and criterion-related standards for shuttle run performance in youth. *Pediatr Exerc Sci* 2012;24:157–69.
172 Mota J, Guerra S, Leandro C. Association of maturation, sex, and body fat in cardiorespiratory fitness. *Am J Hum Biol* 2002;14:707–12.
173 Ribeiro RR, Santos KD, Carvalho WCG, *et al*. Aerobic fitness and biological and sociodemographic indicators in female school children. *Rev Bras Cineantropom Desempenho Hum* 2013;15:448–57.
174 Hobold E. *Indicadores de aptidão física relacionada à saúde de crianças e adolescentes do município de Marechal Cândido Rondon* [dissertation]; Paraná, Brazil: Universidade Federal de Santa Catarina, 2003.
175 Pieta S. *Estudio de la aptitud física de una muestra de la población escolar del estado de Paraná mediante la bateria Eurofit* [dissertation]. León, Brazil: Universidad de León, 2000.
176 Shahin A. Ying and yang of body composition assessment. *Chin J Integr Med* 2011;17:675–9.
177 Tinazci C, Emíroğlu O. Assessment of physical fitness levels, gender and age differences of rural and urban elementary school children. *Turkiye Klinikleri J Med Sci* 2010;30:1–7.
178 Kim H-B, Stebbins CL, Chai J-H, *et al*. Taekwondo training and fitness in female adolescents. *J Sports Sci* 2011;29:133–8.
179 Welk GJ, Saint-Maurice PF, Allums-Featherston K, *et al*. Design and Evaluation of the NFL PLAY 60 FITNESSGRAM Partnership Project. *Res Q Exerc Sport* 2016;87:1–13. .
180 Carrel AL, Bowser J, White D, *et al*. Standardized childhood fitness percentiles derived from school-based testing. *J Pediatr* 2012;161:120–4.
181 Liu W, Zillifro TD, Nichols RA. Tracking of health-related physical fitness for middle school boys and girls. *Pediatr Exerc Sci* 2012;24:549–62.
182 Welk GJ, De Saint-Maurice Maduro PF, Laurson KR, *et al*. Field evaluation of the new FITNESSGRAM® criterion-referenced standards. *Am J Prev Med* 2011;41 (Suppl 2):S131–42.
183 Beets MW, Pitetti KH, Cardinal BJ. Progressive aerobic cardiovascular endurance run and body mass index among an ethnically diverse sample of 10-15-year-olds. *Res Q Exerc Sport* 2005;76:389–97.
184 Beets MW, Pitetti KH. A comparison of shuttle-run performance between mid-western youth and their national and international counterparts. *Pediatr Exerc Sci* 2004;16:94–112.
185 Welk GJ, Schaben JA, Shelley M. Physical activity and physical fitness in children schooled at home and children attending public schools. *Pediatr Exer Sci* 2004;16:310–23.
186 Lloyd LK, Bishop PA, Walker JL, *et al*. The influence of body size and composition on FITNESSGRAM® test performance and the adjustment of FITNESSGRAM® test scores for skinfold thickness in youth. *Meas Phys Educ Exerc Sci* 2003;7:205–26.
187 Chun DM, Corbin CB, Pangrazi RP. Validation of criterion-referenced standards for the mile run and progressive aerobic cardiovascular endurance tests. *Res Q Exerc Sport* 2000;71:125–34.
188 Wolford N. *The difference in physical fitness levels of fifth graders according to socioeconomic groups and genders* [dissertation]. Ann Arbor, MI: University Microforms International, 1998.
189 Mahar MT, Rowe DA, Parker CR, *et al*. Criterion-referenced and norm-referenced agreement between the mile run/walk and PACER. *Meas Phys Educ Exerc Sci* 1997;1:245–58.
190 Sauka M, Priedite IS, Artjuhova L, *et al*. Physical fitness in Northern European youth: reference values from the Latvian physical health in youth study. *Scand J Public Health* 2011;39:35–43.
191 Cossio-Bolaños MA, Arruda M. Propuesta de valores normativos para la evaluación de la aptitud física en niños de 6 a 12 años de Arequipa, Perú. *Rev Med Herediana* 2009;20:206–12.
192 Galaviz KI, Tremblay MS, Colley R, *et al*. Associations between physical activity, cardiorespiratory fitness, and obesity in Mexican children. *Salud Pública Méx* 2012;54:463–9.
193 Ortega FB, Ruiz JR, Labayen I, *et al*. Health inequalities in urban adolescents: role of physical activity, diet, and genetics. *Pediatrics* 2014;133:e884–95.
194 Cooper AR, Goodman A, Page AS, *et al*. Objectively measured physical activity and sedentary time in youth: the International children's accelerometer database (ICAD). *Int J Behav Nutr* 2015;15:113.
195 Guthold R, Cowan MJ, Autenrieth CS, *et al*. Physical activity and sedentary behavior among schoolchildren: a 34-country comparison. *J Pediatrics* 2010;157:43–9.
196 Currie C, Zanotti C, Morgan D, *et al*, eds. Social determinants of health and well-being among young people. Health behaviour in school-aged children (HSBC) study: international report from the 2009/2010 survey. Copenhagen, WHO Regional Office for Europe, 2012: Health Policy for Children and Adolescents, No. 6.
197 Malina R, Bouchard C, Bar-Or O, eds. *Growth, maturation, and physical activity*. 2nd edn. Champaign, IL: Human Kinetics, 2004.
198 Sallis JF, Owen N. Ecological models of health behaviour. In: Glanz K, Lewis FM, Rimer BK, eds. *Health behaviour and health education: theory, research, and practice*. 3rd edn. San Francisco, CA: Jossey-Bass, 2002:462–84.
199 Parikh T, Stratton G. Influence of intensity of physical activity on adiposity and cardiorespiratory fitness in 5-18-year-olds. *Sports Med* 2011;41:477–88.
200 Welk GJ, Corbin CB, Dale D. Measurement issues in the assessment of physical activity in children. *ResQ Exerc Sport* 2000;71(2 Suppl):S59–73.
201 Ekelund U, Tomkinson GR, Armstrong N. What proportion of youth are physically active? Measurement issues, levels and recent time trends. *Br J Sports Med* 2011;45:859–65.

202 World Health Organization. Global School-based Student Health Survey (GSHS) website. http://www.who.int/chp/gshs/en

203 Roberts C, Currie C, Samdal O, *et al*. Measuring the health and health behaviours of adolescents through cross-national survey research: recent developments in the Health Behaviour in School-aged Children (HSBC) study. *J Pub Health* 2007;15:179–86.

204 Hallal PC, Andersen LB, Bull FC, *et al*. Global physical activity levels: surveillance progress, pitfalls, and prospects. *Lancet* 2012;380:247–57.

205 Katzmarzyk PT, Barreira TV, Broyles ST, *et al*. Physical activity, sedentary time, and obesity in an international sample of children. *Med Sci Sports Exerc* 2015;47:2062–9.

206 United Nations. United Nations Expert Group Meeting on Population Distribution, Urbanization, Internal Migration & Development, New York, 21–23 January 2008. New York: United Nations, 2008.

207 Omran AR. The epidemiological transition. A theory of the epidemiology of population change. *Milbank Mem Fund Q* 1971;49:509–38.

208 Katzmarzyk PT, Mason C. The physical activity transition. *J Phys Act Health* 2009;6:269–80.

209 Viner RM, Ozer EM, Denny S, *et al*. Adolescence and the social determinants of health. *Lancet* 2012;379:1641–52.

210 Elgar FJ, Pförtner TK, Moor I, *et al*. Socioeconomic inequalities in adolescent health 2002-2010: a time-series analysis of 34 countries participating in the Health Behaviour in School-aged Children study. *Lancet* 2015;385:2088–95.

211 Lussier L, Buskirk ER. Effects of an endurance training regimen of assessment of work capacity in prepubertal children. *Ann N Y Acad Sci* 1977;301:734–47.

212 Krahenbuhl GS, Pangrazi RP, Chomokos EA. Aerobic responses of young boys to submaximal running. *Res Q* 1979;50:413–21.

213 Sjödin B. The relationship among running economy, aerobic power, and onset of blood lactate accumulation in young boys (11-15 years). In: Komi PV, ed. *Exercise and sport biology*. Champaign, IL: Human Kinetics, 1982:57–60.

214 Berenson GS, Srinivasan SR, Boa W, *et al*. Association between multiple cardiovascular risk factors and atherosclerosis in children and young adults. The Bogalusa Heart Study. *N Engl J Med* 1998;338:1650–6.

215 Lavie CJ, De Schutter A, Milani RV. Health obese versus unhealthy lean: the obesity paradox. *Nat Rev Endocrinol* 2015;11:55–62.

216 Myers J, McAuley P, Lavie CJ, *et al*. Physical activity and cardiorespiratory fitness as major markers of cardiovascular risk: their independent and interwoven importance to health status. *Prog Cardiovasc Dis* 2015;57:306–14.

View publication stats
