# Supplementary material for: Associations between fundamental movement skills and accelerometer-measured physical activity in Chinese children: the mediating role of cardiorespiratory fitness
Source: PeerJ. 2024 Jun 24;12:e17564. doi: 10.7717/peerj.17564 (PMC11210481; doi:10.7717/peerj.17564)
Supplement: Supplemental Information 6 [file peerj-12-17564-s006.docx]

STROBE Statement—Checklist of items that should be included in reports of ***cross-sectional studies***

**Item**

**No Recommendation inc OR excl**

| **Title and abstract** | | 1 | | | (*a*) Indicate the study’s design with a commonly used term in the title or the abstract | inc in line 28 of manuscript |  |
| --- | --- | --- | --- | --- | --- | --- | --- |
|  |  |  |  |  | (*b*) Provide in the abstract an informative and balanced summary of what was done and what was found | inc in line 28-38 |  |
| **Introduction** | | | | | |  |  |
| Background | | 2 | | | Explain the scientific background and rationale for the investigation being reported | inc in line 45 -54 |  |
| Objectives | | 3 | | | State specific objectives, including any prespecified hypotheses | inc in line 101 -103 |  |
| **Methods** | | | | | |  |  |
| Study design | | 4 | | | Present key elements of study design early in the paper | inc in line 107 -124 |  |
| Setting | | 5 | | | Describe the setting, locations, and relevant dates, including periods of recruitment, exposure, follow-up, and data collection | inc in line 107 -124 |  |
| Participants | | 6 | | | (*a*) Give the eligibility criteria, and the sources and methods of selection of participants | inc in line 107-124 |  |
| Variables | | 7 | | | Clearly define all outcomes, exposures, predictors, potential confounders, and effect modifiers. Give diagnostic criteria, if applicable | inc in line 168-172 |  |
| Data sources/ | | 8* | | | For each variable of interest, give sources of data and details of methods of | inc in line 126 -157 |  |
| measurement | |  | | | assessment (measurement). Describe comparability of assessment methods if there is more than one group |  |  |
| Bias | | 9 | | | Describe any efforts to address potential sources of bias | excl |  |
| Study size | | 10 | | | Explain how the study size was arrived at | excl |  |
| Quantitative variables | | 11 | | | Explain how quantitative variables were handled in the analyses. If applicable, describe which groupings were chosen and why | inc in line 190 -194 |  |
| Statistical methods | | 12 | | | (*a*) Describe all statistical methods, including those used to control for confounding | inc in line 181 -206 |  |
|  |  |  |  |  | (*b*) Describe any methods used to examine subgroups and interactions | inc in line 183 -185 and 190-192 |  |
|  |  |  |  |  | (*c*) Explain how missing data were addressed | inc in line 122 -124 |  |
|  |  |  |  |  | (*d*) If applicable, describe analytical methods taking account of sampling strategy | inc in line 107 -112 |  |
|  |  |  |  |  | (*e*) Describe any sensitivity analyses | excl |  |
| **Results** | | | | | |  |  |
| Participants | | 13* | | | (a) Report numbers of individuals at each stage of study—eg numbers potentially eligible, examined for eligibility, confirmed eligible, included in the study,  completing follow-up, and analysed | inc in line 107 -124 |  |
|  |  |  |  |  | (b) Give reasons for non-participation at each stage | inc in line 107 -124 |  |
|  |  |  |  |  | (c) Consider use of a flow diagram | excl |  |
| Descriptive data | | 14* | | | (a) Give characteristics of study participants (eg demographic, clinical, social) and information on exposures and potential confounders | inc in line 209 -211 |  |
|  |  |  |  |  | (b) Indicate number of participants with missing data for each variable of interest | inc in line 122 -124 |  |
| Outcome data | | 15* | | | Report numbers of outcome events or summary measures | inc in line 215 -246 |  |
| Main results | | 16 | | | (*a*) Give unadjusted estimates and, if applicable, confounder-adjusted estimates and their precision (eg, 95% confidence interval). Make clear which confounders were adjusted for and why they were included | excl |  |
|  |  |  |  |  | (*b*) Report category boundaries when continuous variables were categorized | excl |  |
|  |  |  |  |  | (*c*) If relevant, consider translating estimates of relative risk into absolute risk for a meaningful time period | excl |  |
| Other analyses | | 17 | | | Report other analyses done—eg analyses of subgroups and interactions, and sensitivity analyses | inc in line 217 -246 |  |
| **Discussion** | | | | | | | |
| Key results | 18 | | Summarise key results with reference to study objectives inc in line 249 -258 | | | | |
| Limitations | 19 | | | Discuss limitations of the study, taking into account sources of potential inc in line 326 -336  bias or imprecision.  Discuss both direction and magnitude of any potential bias | | | |
| Interpretation | 20 | | | Give a cautious overall interpretation of results considering objectives, excl  limitations, multiplicity of analyses, results from similar studies,  and other relevant evidence | | | |
| Generalisability | 21 | | | Discuss the generalisability (external validity) of the study results excl | | | |
| **Other information** | | | | | | | |
| Funding | 22 | | | Give the source of funding and the role of the funders for the present study inc in line 369 -371  and, if applicable,for the original study on which the present article is based | | | |

*Give information separately for exposed and unexposed groups.

**Note:** An Explanation and Elaboration article discusses each checklist item and gives methodological background and published examples of transparent reporting. The STROBE checklist is best used in conjunction with this article (freely available on the Websites of PLoS Medicine at <http://www.plosmedicine.org/>, Annals of Internal Medicine at

<http://www.annals.org/>, and Epidemiology at http://www.epidem.com/). Information on the STROBE Initiative is available at www.strobe-statement.org.
